# Thermosensory predictive coding underpins an illusion of pain

**DOI:** 10.1126/sciadv.adq0261

**Published:** 2025-03-12

**Authors:** Jesper Fischer Ehmsen, Niia Nikolova, Daniel Elmstrøm Christensen, Leah Banellis, Rebecca A. Böhme, Malthe Brændholt, Arthur S. Courtin, Camilla E. Krænge, Alexandra G. Mitchell, Camila Sardeto Deolindo, Christian Holm Steenkjær, Melina Vejlø, Christoph Mathys, Micah G. Allen, Francesca Fardo

**Affiliations:** ^1^ Center of Functionally Integrative Neuroscience (CFIN), Department of Clinical Medicine, Aarhus University, Aarhus, Denmark.; ^2^ BioMedical Design, Department of Clinical Medicine, Aarhus University, Aarhus, Denmark.; ^3^ Institute of Neuroscience (IoNS), Université catholique de Louvain, Brussels, Belgium.; ^4^ Department of Neurology, Aalborg University Hospital, Aalborg, Denmark.; ^5^ Interacting Minds Center (IMC), Aarhus University, Aarhus, Denmark.; ^6^ Cambridge Psychiatry, University of Cambridge, Cambridge, UK.; ^7^ Danish Pain Research Center, Department of Clinical Medicine, Aarhus University, Aarhus, Denmark.

## Abstract

The human brain has a remarkable ability to learn and update its beliefs about the world. Here, we investigate how thermosensory learning shapes our subjective experience of temperature and the misperception of pain in response to harmless thermal stimuli. Through computational modeling, we demonstrate that the brain uses a probabilistic predictive coding scheme to update beliefs about temperature changes based on their uncertainty. We find that these expectations directly modulate the perception of pain in the thermal grill illusion. Quantitative microstructural brain imaging further revealed that individual variability in computational parameters related to uncertainty-driven learning and decision-making is reflected in the microstructure of brain regions such as the precuneus, posterior cingulate gyrus, cerebellum, as well as basal ganglia and brainstem. These findings provide a framework to understand how the brain infers pain from innocuous thermal inputs, with important implications for the etiology of thermosensory symptoms under chronic pain conditions.

## Teaser

Computational modeling reveals how uncertainty transforms harmless stimuli into perceptions of pain.

## INTRODUCTION

The ability to adapt to environmental changes and learn in the face of uncertainty is critical for generating precise and flexible responses to a wide range of stimuli. In the context of thermosensation and nociception, such adaptability allows us to effectively detect temperature shifts and avert potential tissue damage, even under conditions of incomplete or ambiguous information. This capability not only is essential for safeguarding our bodily integrity but also facilitates our interaction with an uncertain environment. Here, we report findings demonstrating that thermosensation relies on precision-weighted expectations and that this extends to complex phenomena such as illusory pain, exemplified by the thermal grill illusion (TGI).

Current knowledge of the thermosensory and thermonociceptive systems predominantly revolves around peripheral sensory mechanisms that transduce innocuous and noxious thermal stimuli into neural signals. This includes landmark discoveries like the TRPV1 and TRPM8 receptors (*1*–*3*). Although these bottom-up mechanisms have been extensively studied, less attention has been devoted to how they integrate with top-down expectations to form our subjective experiences of temperature and pain. Perception in these domains is not solely the output of isolated afferent channels but is heavily influenced by prior beliefs and expectations (*4*–*7*). In this context, the TGI presents a notable case in which the simultaneous presentation of innocuous warm and cold stimuli can evoke illusory burning sensations (*8*–*13*). This illusion is fascinating because it cannot be adequately explained by the physical characteristics of the stimuli alone. Instead, it arises from a dynamic interplay between sensory processing and cognitive factors, collectively shaping how temperature and pain are perceived. Although neuroimaging studies have mapped out brain regions involved in the TGI, such as the anterior cingulate cortex, thalamus, cerebellum, hippocampus, and parietal regions (*14*–*16*), the influence of expectations on this phenomenon remains an open and largely underexplored question.

Associative learning plays a fundamental role in the perception of pain and its modulation by expectation, enabling the development of adaptive behaviors that protect us from potential harm. Substantial progress has been made in understanding these processes through the computational neuroscience of predictive coding (*17*–*26*) and reinforcement learning (*22*, *27*). For instance, it has been shown that participants learn about painful stimuli in a manner that is consistent with Bayesian principles (*27*, *28*), and pain-prediction errors have been mapped to key brain areas involved in pain-related processing, including the insula and brainstem (*19*, *29*, *30*). A key contribution of this work was the recognition that expectation-related modulation of pain, such as nocebo and placebo effects (*31*–*37*), are grounded in the weighting of pain prediction errors by their uncertainty or inverse precision (*17*, *22*, *38*–*40*). To date, it is unknown if these principles similarly explain innocuous thermosensory perception and illusions of pain. An intriguing possibility is that the TGI may stem from thermosensory predictive coding, where increased uncertainty about upcoming stimulus temperatures enhances the perception of pain.

In this study, we apply computational methods to reveal how expectations and their associated uncertainty influence both innocuous thermosensation and the TGI. We further used high-resolution quantitative magnetic resonance imaging (qMRI) to identify how interindividual variations in the brain microstructure are associated with computational fingerprints of thermosensory learning. The brain’s microstructural properties, including variations in myelination and iron concentration, play a crucial role in shaping individual differences in thermosensation and pain perception. Structural variations, particularly within pain-related pathways, likely influence how sensory input is processed and interpreted. Understanding these neurobiological features offers important insights into the mechanisms behind such perceptual differences, enhancing our understanding of the biological factors that contribute to the variability in thermosensory and pain responses among individuals (*41*).

To this aim, we conducted an experiment in 267 participants who completed a probabilistic thermosensory learning (PTL) task, in which we strategically embedded simultaneous cold and warm stimuli to induce the TGI within the learning sequence. This experimental approach offers a comprehensive analysis of the role of expectations in thermosensory learning and provides a unique opportunity to test the hypothesis that the uncertainty of thermal expectations plays a crucial role in the perception of illusory pain. Our results provide a compelling example of how uncertainty contributes to the misinterpretation of non-nociceptive stimuli as painful, offering potential insights into symptoms of neuropathic and nociplastic pain conditions (*42*, *43*).

## RESULTS

**Fig. 1.**
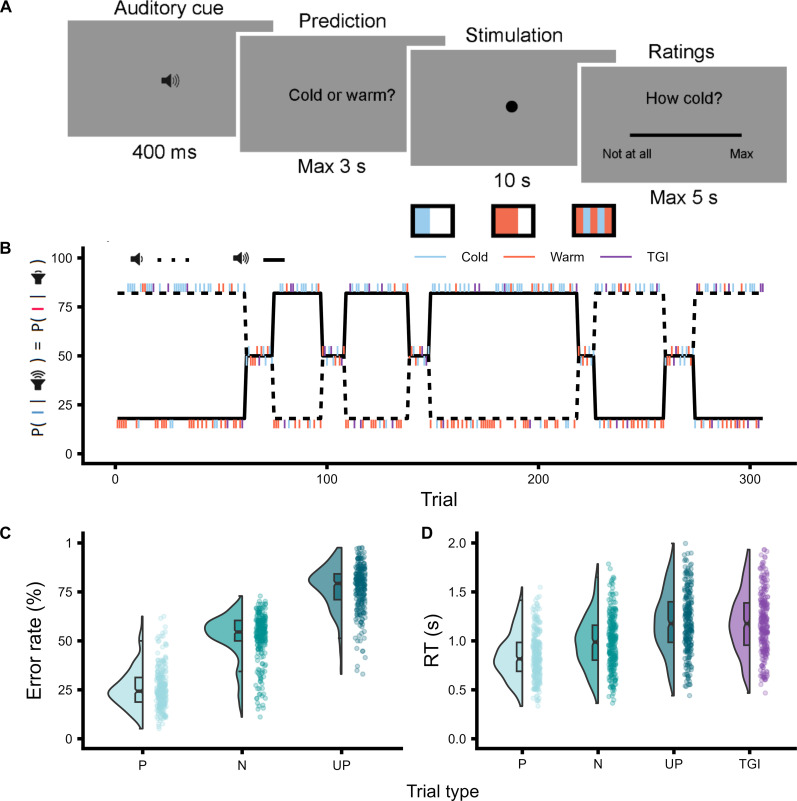
Thermosensory learning: Experimental design and behavioral measures. (**A**) Trial structure depicting the sequence of events within each trial: auditory cue presentation, prediction of the forthcoming stimulation quality as either cold or warm, delivery of the thermal stimulation [cold, warm, or thermal grill illusion (TGI)], and visual analog scale (VAS) ratings of cold, warm, and burning sensations. All three ratings were completed for a given stimulus. (**B**) Time course of cue-stimulus contingencies throughout the experiment, varying across three levels of cue-stimulus association probabilities set at 82, 50, and 18%. (**C**) Comparison of error rates for participants’ predictions of the forthcoming stimulation quality across predicted (P), neutral (N), and unpredicted (UP) innocuous thermosensory trials. (**D**) Comparison of response times (RT) in the trial following predicted (P), neutral (N), and unpredicted (UP) thermosensory stimuli, as well as TGI stimuli, demonstrating postprediction error slowing.

To quantify the relationship between learned expectations and thermosensation, we tested the PTL task (Fig. 1A) in 267 healthy individuals. The PTL integrates key features of reversal learning tasks in other sensory domains (*44*–*47*), in which participants must dynamically update sensory predictions in response to varying uncertainty. In each PTL trial, participants heard auditory cues consisting of high or low tones that predicted whether the forthcoming stimulus would be cold or warm. Critically, these cue-stimulus pairings shifted unpredictably over time, requiring participants to continuously relearn their associative mappings. Cue-stimulus associations varied according to blocks of longer, more stable periods in which reversals were less likely, and shorter, more variable periods in which transitions occurred more frequently (Fig. 1B). Innocuous cool and warm trials were pseudorandomly interspersed with ambiguous stimuli. These ambiguous stimuli were the simultaneous presentation of the same objective temperatures as those used in the innocuous cold and warm trials, in an alternated spatial configuration. This method of stimulus presentation is known to elicit burning pain sensations referred to as the TGI (*8*–*13*). On each trial, participants made a binary prediction response, indicating whether they expected an upcoming cold or warm stimulus. In a subset of trials, they subsequently provided visual analog scale (VAS) ratings reflecting their perceived levels of cold, warm, and burning sensations. This design allowed us to isolate how uncertainty in learned cue-stimulus associations affects the perception of ambiguous thermal stimuli, such as those that produce the TGI. By manipulating the frequency and stability of cue-stimulus reversals, we could precisely quantify how top-down expectations, and their associated uncertainty, modulated the quality and intensity of this pain illusion.

### Behavior

#### Error rates and response times are modulated by thermosensory learning

To evaluate participants’ learning of cue-stimulus associations, we analyzed error rates for predicted, neutral, and unpredicted innocuous thermosensory stimuli (Fig. 1C and table S1). Predicted and unpredicted stimuli were defined based on the participants’ trial-by-trial predictions (i.e., whether they predicted a cold or a warm sensation) in blocks where the nominal probability of a specific cue-stimulus association was 82 and 18%, respectively. Neutral trials referred to nonpredictive blocks where a cue predicted a particular stimulus with a 50% probability. This analysis confirmed that the probability of cue-stimulus association robustly modulated expectations such that participants’ prediction accuracy was highest for predicted trials compared to both neutral [β = −1.34, 95% confidence interval (CI) = [−1.38; −1.29], *P* < 0.0001] and unpredicted trials (β = −2.26, 95% CI = [−2.31; −2.21], *P* < 0.0001).

As further evidence of successful learning, we observed postprediction error slowing, indicated by reduced response times on trials following association violations (Fig. 1D and table S2). Our findings showed that response times were increasingly slowed following neutral (β = 0.15, 95% CI = [0.13; 0.16], *P* < 0.0001), unpredicted (β = 0.31, 95% CI = [0.3; 0.32], *P* < 0.0001), and TGI stimuli (β = 0.34, 95% CI = [0.32; 0.35], *P* < 0.0001), compared to predicted stimuli. Together, these results serve as a model-free positive control, confirming that participants effectively learned and incorporated cue-stimulus relationships into their thermosensory predictions.

#### Stimulus-specific effects on Thermosensory and burning ratings

**Fig. 2.**
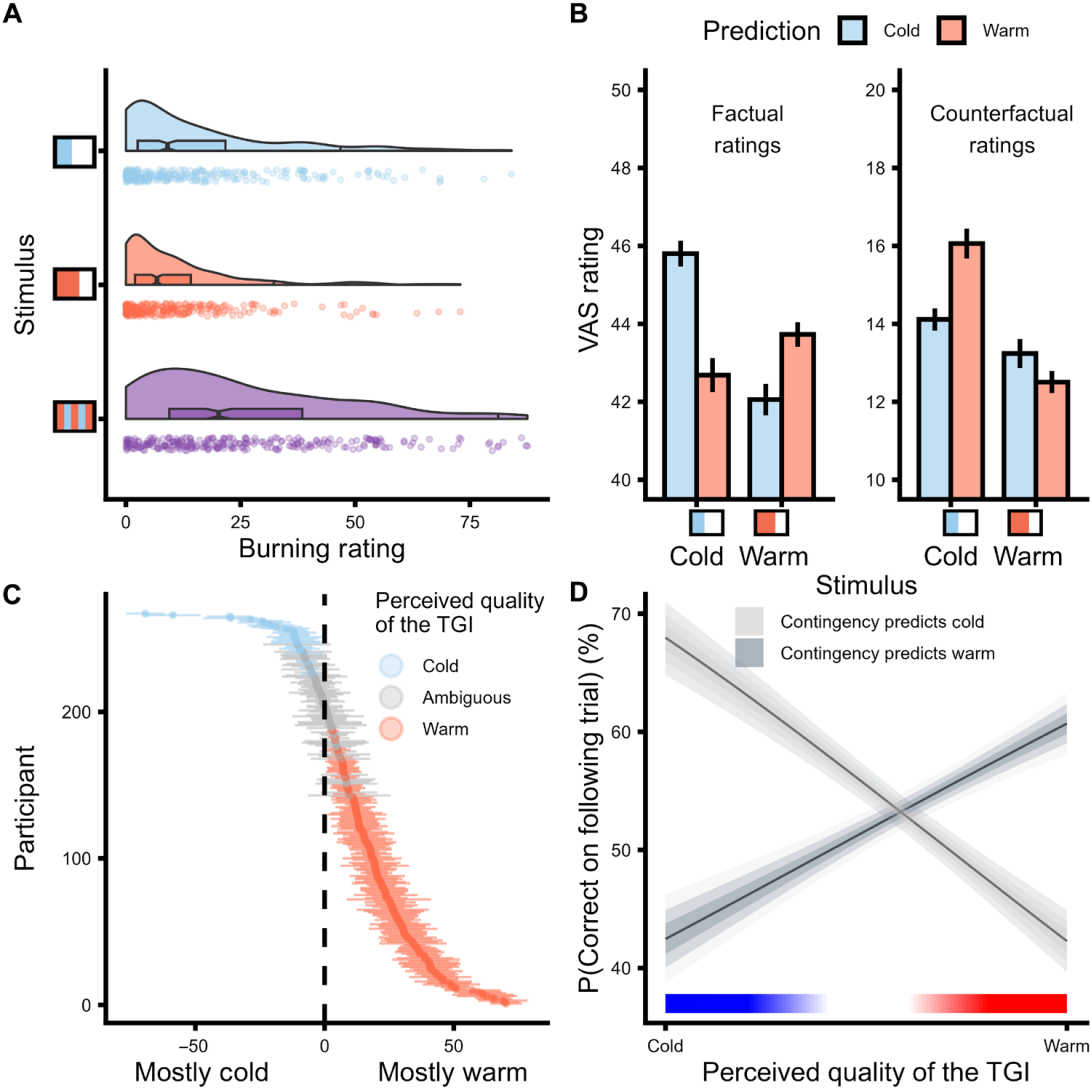
Thermosensory ratings and thermal grill illusion (TGI) perception. (**A**) Visual analog scale (VAS) burning ratings for cold, warm, and TGI stimuli, illustrating a key feature of the TGI as an illusion of pain. (**B**) Effects of participants’ expectations on VAS ratings for innocuous cold and warm stimuli, showing that expectations modulated these sensations (means ± 2 SEM). (**C**) Within-subject consistency of TGI perception as mostly cold (blue), ambiguous (gray), or mostly warm (red). Thermal ambiguity in this context signified that participants perceived the TGI trials as equally warm and cold. The *y* axis depicts each individual participant, whereas the *x* axis represents the ratio of perceived coldness to warmth for TGI stimuli (means ± 2 SEM). (**D**) Relationship between perceived TGI quality and learning (i.e., error rates in the trials that followed TGI stimulation), demonstrating that TGI trials reinforced cue-stimulus contingencies based on the perceived TGI quality.

To evaluate the effectiveness of cold, warm, and TGI stimuli, we predicted subjective ratings using linear mixed effects models incorporating a zero-one inflated beta regression approach (Supplementary Note). The TGI is characterized by an enhanced perception of heat and the elicitation of burning sensations when innocuous cold and warm stimuli are combined, which does not occur when these stimuli are applied individually (Fig. 2A and tables S3 to S5). In line with heat enhancement, TGI stimuli were rated as significantly less cold than innocuous cold stimuli (β = −0.39, 95% CI = [−0.41; −0.37], *P* < 0.0001) but warmer than innocuous warm alone (β = 0.18, 95% CI = [0.17; 0.2], *P* < 0.0001). Furthermore, in line with the elicitation of illusory pain, the concurrent application of cold and warm stimuli during TGI produced significantly greater burning sensations than when either cold (β = −0.45, 95% CI = [−0.48; −0.43], *P* < 0.0001) or warm stimuli (β = −0.65, 95% CI = [−0.68; −0.63], *P* < 0.0001) were applied individually. Together, these findings confirm that innocuous thermosensory stimuli were perceived in a veridical manner, and the TGI manipulation effectively induced illusory heat and burning sensations.

#### Innocuous thermosensation is shaped by expectations

To investigate the impact of learned expectations on innocuous thermosensory experiences, we analyzed participants’ reported levels of both cold and warm sensations for predicted and unpredicted stimuli (Fig. 2B and table S6). For each stimulus, participants provided ratings for factual (e.g., coldness of a cold stimulus) and counterfactual qualities (e.g., warmth of a cold stimulus) of their sensations. We found a robust three-way interaction between the stimulation quality, the participants’ prediction on a trial-by-trial basis, and the rating type (β = 0.33, 95% CI = [0.26; 0.40], *P* < 0.0001). Considering the factual ratings, predicted cold stimuli were rated as colder than predicted warm stimuli (β = −0.10, 95% CI = [−0.12; −0.07], *P* < 0.0001), and predicted warm stimuli were rated as warmer than predicted cold stimuli (β = −0.06, 95% CI = [−0.08; −0.04], *P* < 0.0001). Conversely, when assessing the counterfactual quality, predicted cold stimuli were rated as less warm compared to predicted warm stimuli (β = 0.12, 95% CI = [0.08; 0.16], *P* < 0.0001), whereas predicted cold stimuli were rated as colder compared to predicted warm stimuli (β = 0.05, 95% CI = [0.01; 0.09], *P* < 0.05). Overall, these results highlight that participants’ thermosensory expectations influenced the perceived intensity of innocuous stimuli.

#### Response times and error rates reflect perceived TGI quality

We hypothesized that the ambiguous nature of TGI trials would either reinforce or counter cue-stimulus associations, depending on the participants’ perception of TGI as primarily warm or cold. For instance, if a participant associates a high tone with a high probability of experiencing a cold stimulus and perceives a TGI stimulus as predominantly warm, they might incorrectly infer a reversal has occurred after hearing a high tone and receiving a TGI stimulus, leading to an erroneous prediction in the subsequent trial. Conversely, if a participant perceives the TGI as predominantly cold, the participant’s correct association would be reinforced, leading to increased likelihood of an accurate prediction in the subsequent trial. To evaluate this hypothesis, we assessed each participant’s perceived TGI quality by computing the ratio of perceived coldness to warmth. In general, participants displayed high self-consistency in evaluating their perception of TGI stimuli as predominantly cold or warm (Fig. 2C).

Our model confirmed our hypothesis, demonstrating that error rates were significantly influenced by the interaction between cue-stimulus association and perceived TGI quality (β = −1.73, 95% CI = [−2.04; −1.42], *P* < 0.0001; Fig. 2D and table S7). Specifically, participants were more likely to respond correctly on the subsequent trial when the contingency and the perceived TGI quality matched (i.e., predicting cold and perceiving TGI as predominantly cold) (β = 1.02, 95% CI = [0.81; 1.24], *P* < 0.0001). Conversely, participants were more likely to make incorrect responses in the following trial when the contingency and the perceived TGI quality diverged (e.g., predicting warm and perceiving TGI as predominantly cold) (β = −0.71, 95% CI = [−0.94; −0.48], *P* < 0.0001). Collectively, in a contingency block that predicted a cold outcome, the odds of making a correct prediction changed substantially depending on whether the TGI was rated as mostly cold versus warm, a difference amounting to an 18.36% change in the probability of a correct answer [14.08, 22.13%]. Complementary effects were observed for response times after TGI stimulation (see Supplementary Results and table S8). In summary, these findings reveal that TGI trials play a crucial role in reinforcing cue-stimulus associations by effectively shaping participants’ thermosensory predictions based on their perceived quality.

### Computational modeling and brain imaging

#### A two-level hierarchical Gaussian Filter model best explained thermosensory learning

**Fig. 3.**
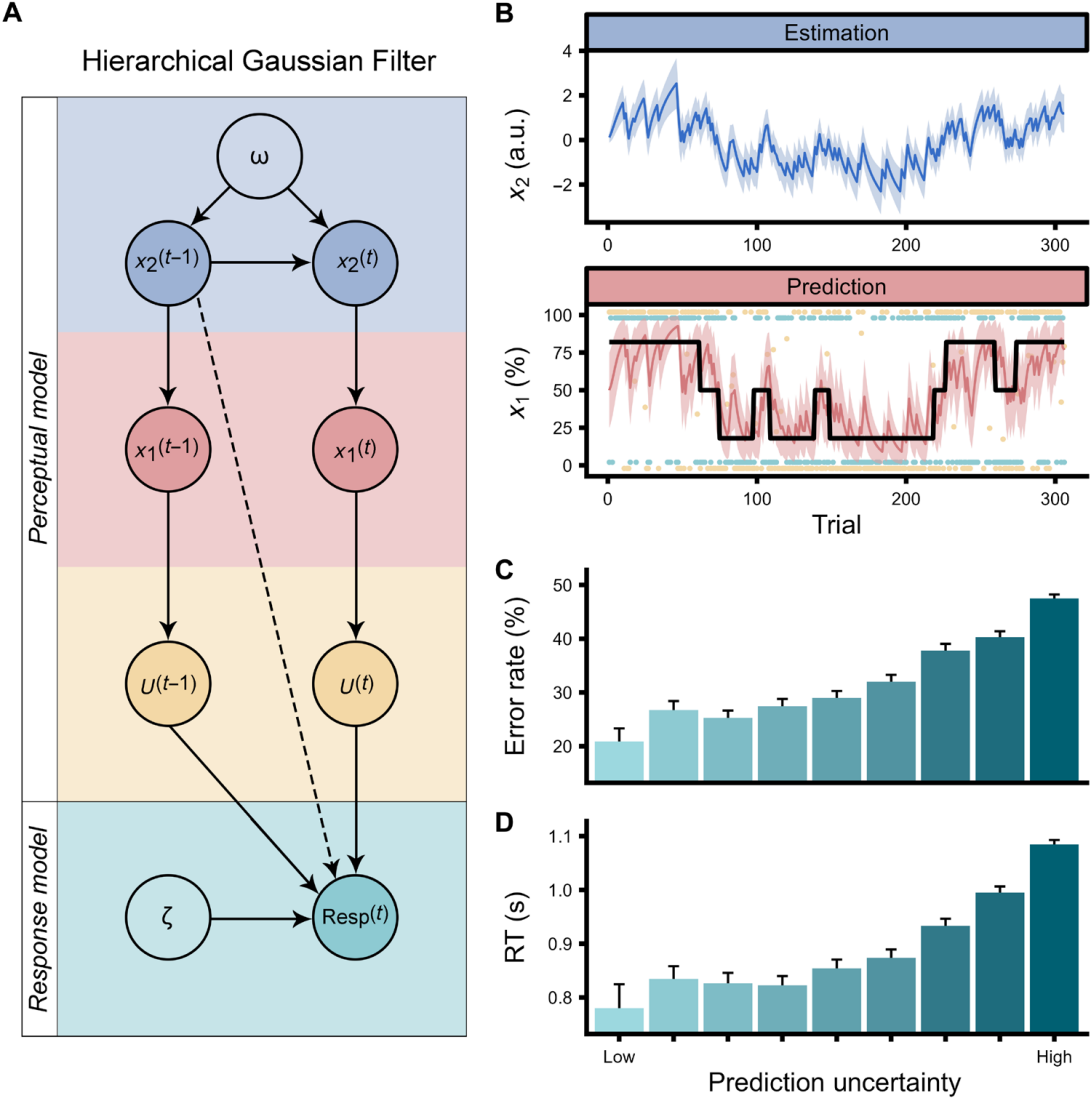
Computational modeling of thermosensation and illusory pain. (**A**) Illustration of the Hierarchical Gaussian Filter (HGF) and its constituent perceptual and response models. Within the perceptual model, two hierarchical levels of trajectories with uncertainties are defined: prediction (*x*_1_) and estimation (*x*_2_). The first level takes the form of a Bernoulli distribution, whereas the second level evolves in time as a Gaussian random walk with step size corresponding to the omega (ω) parameter. The response model converts the continually updated perceptual belief to a probability of answering through the inverse decision temperature zeta (ζ) through a logistic sigmoid transformation. *U* are observed values representing the cue-stimulus association mappings, and dashed line depicts mediation through model inversion. (**B**) Example of a single participants’ prediction and estimation trajectories together with their respective uncertainties. When considering the prediction trajectory, the black thick line represents the actual contingency probabilities. The trial-by-trial participant’s responses (i.e., predictions) are depicted by green points, where the value of one corresponds to the prediction of a cold stimulus and the value of zero corresponds to a prediction of a warm stimulus. The contingency space is represented by yellow dots, where values of zero represent low tone-cold and high tone-warm associations, and values of one represent low tone-warm and high tone-cold associations. Intermediate values represent trials in which the stimulus was simultaneously cold and warm [i.e., thermal grill illusion (TGI)]. a.u., arbitrary units. Prediction uncertainty strongly modulated both (**C**) error rates and (**D**) response times needed to provide a prediction about the upcoming stimulus, validating the response model. Prediction uncertainty is presented here as discretized into nine bins.

We used the Hierarchical Gaussian Filter (HGF) (*48*, *49*) to analyze learning trajectories across two hierarchical levels of belief (Fig. 3). We estimated mean and uncertainty values for beliefs about an upcoming stimulus given a cue (i.e., predictions) and beliefs regarding the strength of cue-outcome associations (i.e., estimations). At the first level (*x*_1_), prediction uncertainty pertains to uncertainty about immediate outcomes. A low prediction uncertainty indicates high confidence in predicting the forthcoming stimulus based on the given cue, whereas a high prediction uncertainty suggests that the participant has not formed a definite prediction of which outcome is most likely. At the second level (*x*_2_), estimation uncertainty quantifies the uncertainty surrounding the reliability of cue-outcome relationships. This level of uncertainty influences the rate at which beliefs about cue-outcome associations are updated. Low estimation uncertainty signifies a strong belief in the consistency of the cue-outcome association, requiring considerable contrary evidence for a belief update. In contrast, high estimation uncertainty means that beliefs regarding the cue-outcome relationship are more malleable and can be adjusted more readily upon encountering disconfirming evidence. Prediction and estimation uncertainty are structured hierarchically, meaning that beliefs at one level are dependent on, or informed by, the beliefs at the upper level (Fig. 3, A and B).

To assess the best-fitting model while accounting for parameter complexity, we compared the two-level HGF with other well-known reinforcement learning models, such as Rescorla-Wagner, Sutton K1, and Pearce-Hall (*50*–*53*) using Bayesian model selection (*54*). We found that the two-level HGF outperformed these models. To validate the robustness of our fitted models, we conducted both parameter and model recovery for the models under consideration (see figs. S1 to S8). Overall, model comparison and cross-validation demonstrated that thermosensory learning is best captured by Bayesian precision-weighted mechanisms that integrate both prediction and estimation uncertainty.

#### Modulation of behavior and perception by uncertainty

**Fig. 4.**
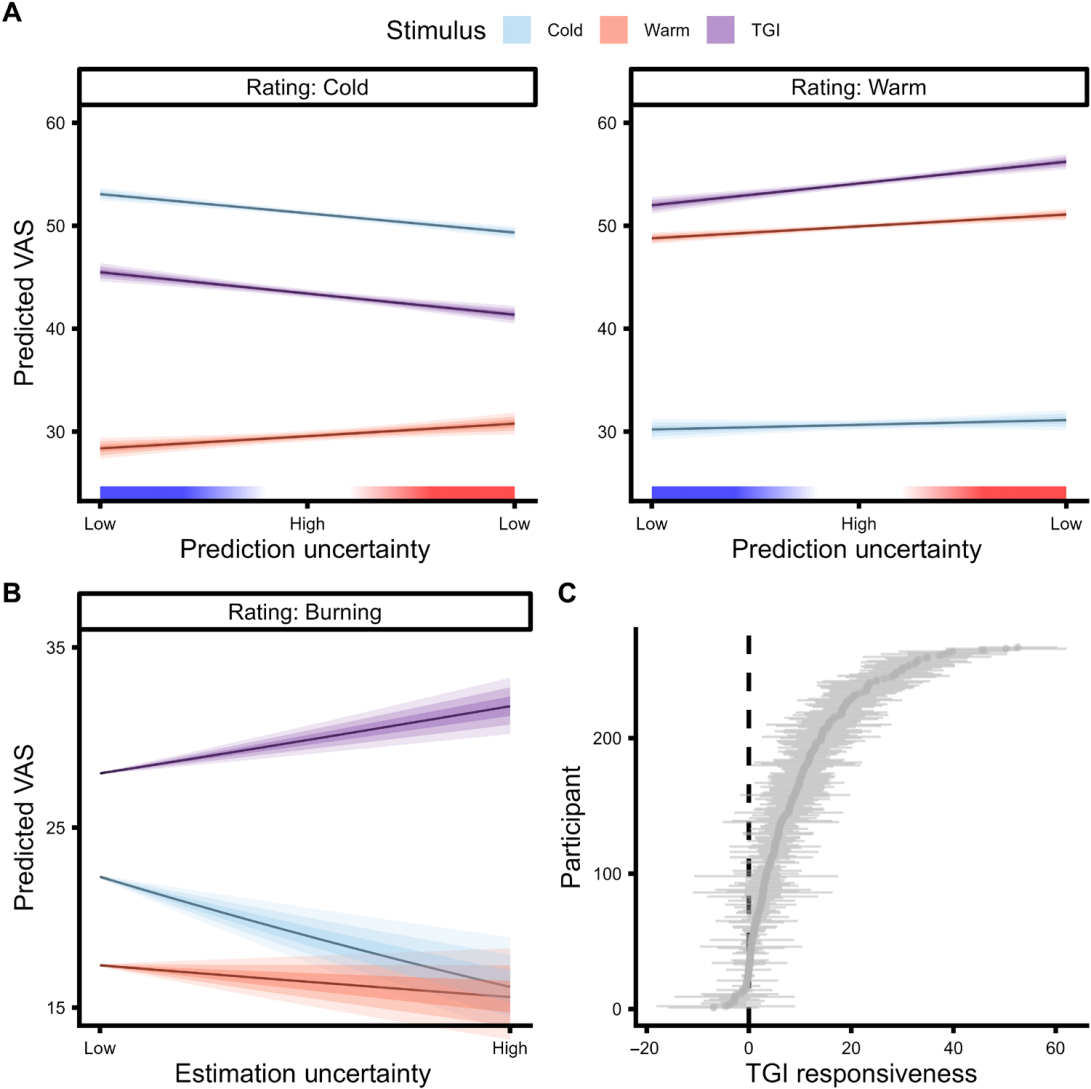
Effects of prediction and estimation uncertainty on veridical thermosensation and illusory pain. (**A**) Impact of prediction uncertainty on thermosensory ratings for cold (blue), warm (red), and thermal grill illusion (TGI) stimuli (purple). The *x* axis represents the precision of the lower-level belief about the forthcoming stimulus. Prediction uncertainty values range from high precision prediction that the stimulus would be warm to high precision predictions that the stimulus would be cold. Intermediate values indicate high prediction uncertainty about the thermal quality of the forthcoming stimulus. The *y* axis indicates the predicted visual analog scale (VAS) ratings (i.e., marginal means) based on zero-one inflated beta (ZOIB) modeling, separately for cold, warm, and burning ratings with the shaded area depicting the 50, 80, and 95% CI on the marginal means. (**B**) Impact of estimation uncertainty on TGI perception. The *x* axis depicts the varying degree of estimation uncertainty from low to high. The *y* axis indicates the predicted burning ratings based on ZOIB modeling, separately for cold (blue), warm (red), and TGI stimuli (purple) with the shaded area depicting the 50, 80, and 95% CI on the marginal means. (**C**) Individual differences in TGI responsiveness. The *y* axis depicts each individual participant, whereas the *x* axis represents the TGI responsiveness (means ± 2 SEM), calculated as the discrepancy between burning ratings for TGI stimuli and the highest burning rating for either innocuous cold or warm stimuli. This approach yielded a continuous scale of TGI responsiveness, spanning from negative values indicative of TGI nonresponders to positive values representing TGI responders.

The impact of uncertainty on behavior and subjective experience was assessed using hierarchical regression analyses. At the lower level, involving prediction uncertainty, precise beliefs notably diminished error rates (β = −4.88, 95% CI = [−5.13; −4.63], *P* < 0.0001; Fig. 3C and table S9) and response times (β = 1.76, 95% CI = [1.69; 1.82], *P* < 0.0001; Fig. 3D and table S10) when participants predicted the quality of a forthcoming stimulus. This effect was also reflected in heightened VAS ratings for the thermosensory quality consistent with participants’ expectations (β = −0.19, 95% CI = [−0.28; −0.09], *P* < 0.0001). Specifically, a stronger belief about a forthcoming cold stimulus resulted in heightened cold ratings (β = 0.15, 95% CI = [0.10; 0.19], *P* < 0.0001) but reduced warm ratings (β = −0.09, 95% CI = [−0.13; −0.05], *P* < 0.0001; Fig. 4A and table S11). Prediction uncertainty also exerted a notable influence on the perceived thermosensory quality of the TGI (Fig. 4A and table S11). Here, precise expectations of cold intensified cold ratings (β = 0.17, 95% CI = [0.11; 0.23], *P* < 0.0001) and reduced warm (β = −0.17, 95% CI = [−0.22; −0.11], *P* < 0.0001) but did not significantly influence burning ratings (β = −0.04, 95% CI = [−0.12; 0.03], *P* = 0.26) during the illusion.

On the other hand, the higher-level estimation uncertainty played a differential role in influencing burning sensations within TGI trials compared to control stimuli (β = 0.04, 95% CI = [0.03; 0.06], *P* < 0.0001) (Fig. 4B and table S12). Whereas precise cold expectations at the lower level were linked to reduced burning sensations, weak or unclear associations between cues and predicted stimuli increased burning ratings compared to both cold (β = −0.11, 95% CI = [−0.14; −0.09], *P* < 0.0001) and warm stimuli (β = −0.06, 95% CI = [−0.09; −0.04], *P* < 0.0001). This indicated that the illusory pain aspect of the TGI was most intense under conditions of ambiguous cue-stimulus mappings or high estimation uncertainty. In essence, although lower-level prediction uncertainty predominantly determined whether the TGI was perceived as more cold or warm, the characteristic burning sensation of the TGI was markedly influenced by higher-level estimation uncertainty. These findings indicate how increased uncertainty regarding forthcoming stimulus temperatures can lead to a distorted perception of innocuous temperatures, manifesting as an aberrant sensation of pain.

#### Cortical myeloarchitecture fingerprints of computational parameters

We conducted an exploratory investigation of the neurobiological underpinnings of interindividual variability in computational parameters: omega (ω) reflecting the speed of adaptation to changing conditions or learning; zeta (ζ) capturing variability in the decision-making process, also known as decision temperature; the uncertainty modulation of TGI index (UMTI) reflecting individual differences in the modulation of TGI burning ratings by high order uncertainty (see Supplementary Note); and TGI responsiveness (Fig. 4C).

**Fig. 5.**
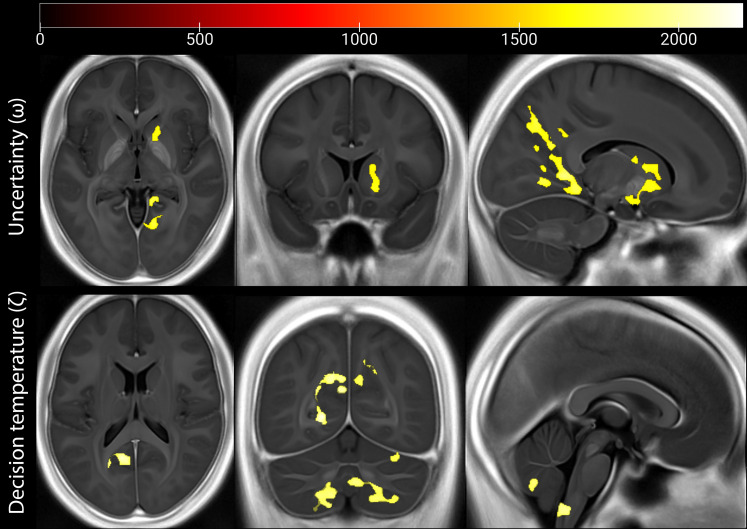
Microstructural brain correlates of computational parameters (omega and zeta contrasts). Using a permutation-based TFCE approach, we identified that iron concentrations (as indexed by R2* maps) in specific cortical, subcortical, and brainstem regions positively correlated with the learning parameter related to uncertainty (ω or omega) and decision temperature (ζ or zeta). The ω parameter reflects the influence of uncertainty on learning, whereas the ζ parameter reflects decision temperature. Heatmaps and color bars indicate TFCE values. For visualization purposes, the thresholded maps are plotted on the average normalized parametric map across the entire sample. The three columns represent axial, coronal, and sagittal views of the brain, from left to right, respectively.

To this aim, we performed whole-brain voxel-based quantification (VBQ) analyses of magnetization transfer (MT), longitudinal relaxation rate (R1), and effective transverse relaxation rate (R2*) weighted maps obtained from 0.8-mm multiparameter mapping (MPM) (Fig. 5 and Tables 1 and 2). MT and R1 serve as indicators of cortical myeloarchitecture, aiding in the identification of myelination levels in gray matter (GM). R2* is influenced by factors such as iron concentration (*55*, *56*). A benefit of this approach is that the VBQ technique yields quantitative measures of the local brain microstructure, which are inherently meaningful and comparable across imaging sites or studies, unlike classical volumetric techniques that derive arbitrary signal units (*56*).

**Table 1.** Microstructural brain correlates of the computational learning parameter omega.

Region	*k*	*p* (FWE)	TFCE	*Z* value	*x*	*y*	*z*
R precuneus	5453	0.015	1999	3.35	4	−54	35
L precuneus					−5	−53	30
R posterior cingulate gyrus	10,683	0.019	1916	3.35	15	−48	4
R precuneus					18	−57	21
L precuneus	2001	0.028	1780	3.09	−4	−88	30
					1	−93	17
R angular gyrus	1023	0.03	1757	3.16	51	−59	15
R middle temporal gyrus					47	−50	11
R caudate	3088	0.034	1713	3.16	13	6	8
R putamen					15	11	−5
R superior temporal gyrus	217	0.039	1667	3.09	43	−46	12
					44	−38	11
L middle frontal gyrus	252	0.043	1628	3.54	−27	59	9
R middle occipital gyrus	351	0.045	1607	3.24	35	−76	45
R angular gyrus					34	−68	46
R superior parietal lobule	155	0.048	1589	3.24	26	−68	44

**Table 2.** Microstructural brain correlates of the computational learning parameter zeta.

Region	*k*	*P* (FWE)	TFCE	*Z* value	*x*	*y*	*z*
L lingual gyrus	7717	0.018	2200	3.09	−19	−59	2
L precuneus					−8	−53	9
R cerebellum	6037	0.028	2008	3.16	10	−55	−54
Brainstem					9	−46	−59
L cerebellum	3281	0.029	2002	3.09	−14	−67	−51
					−40	−55	−54
L cerebellum	1764	0.035	1919	3.09	−34	−46	−28
L fusiform gyrus					−28	−43	−18
R precuneus	1290	0.039	1880	3.16	13	−66	41
					8	−66	28
R lingual gyrus	684	0.042	1844	3.04	13	−41	−6
R cerebellum					12	−37	−14
R cerebellum	260	0.043	1829	3.16	31	−65	−23
R posterior cingulate gyrus	513	0.045	1817	2.95	10	−48	6
L lingual gyrus	336	0.047	1798	3.04	−26	−58	−4
					−30	−50	−5

Using a permutation-based threshold-free cluster enhancement (TFCE) approach (*57*), we identified significant correlations between computational parameters and individual variation in iron concentration in cortical, subcortical, and brainstem regions. The ω parameter showed significant positive correlations with iron concentration variability in several key regions (Table 1). These included the right precuneus [*k* = 5453 voxels, *P*(FWE) = 0.015], right posterior cingulate gyrus [*k* = 10,683 voxels, *P*(FWE) = 0.019], right caudate [*k* = 3088 voxels, *P*(FWE) = 0.034], and right angular gyrus [*k* = 1023 voxels, *P*(FWE) = 0.03]. The implication of the microstructural variability of these regions in thermosensory associative learning profiles is consistent with previous studies linking their function to uncertainty processing, pain modulation, and cognitive control. The ζ parameter was positively correlated with iron concentrations in several brain regions (Table 2), including the left lingual gyrus [*k* = 7717 voxels, *P*(FWE) = 0.018], right cerebellum and brainstem [*k* = 6037 voxels, *P*(FWE) = 0.028], as well as right precuneus [*k* = 1290 voxels, *P*(FWE) = 0.039]. For consistency with prior VBQ studies, we also conducted a classical cluster-based analysis and found additional results linking the microstructure of the inferior frontal gyrus and basolateral amygdala to individual variability in UMTI (see Supplementary Note). Together, these results shed light on the microstructural fingerprints associated with learning and decision-making within the context of thermosensation and pain illusions.

## DISCUSSION

We demonstrate that bottom-up sensory processes and top-down expectations interact to shape the perception of innocuous thermosensation and the TGI, providing a computational perspective on how the brain’s interpretation of innocuous thermal stimuli can paradoxically lead to pain. Through computational modeling, we have defined a mechanistic framework where hierarchical levels of predictions govern both the perceived quality of thermosensory inputs and the intensity of illusory pain. At the lower level, the immediate predictions about an upcoming sensory stimulus modulate the perceived thermosensory quality. This modulation aligns closely with the predicted outcome, with the degree of influence scaled by the prediction’s uncertainty. In other words, more uncertain predictions result in less pronounced effects on temperature perception. At the higher level, the model encapsulates beliefs about cue-outcome associations, where greater uncertainty in these associations is found to amplify the experience of illusory pain, as evidenced by the increased burning sensations during the TGI. This framework highlights that the perception of pain or burning during the TGI arises from the dynamic interplay between the brain’s sensory processing and its predictive mechanisms.

We further defined computationally based metrics of thermosensory learning, decision-making, and TGI responsivity and related such parameters to brain microstructural properties. Using high-resolution multiparameter maps indexing myelination and iron concentration, we identified significant relationships between the computational parameters omega (ω) and zeta (ζ) and the iron concentration of brain regions involved in pain modulation, sensory integration, and cognitive control.

The ω parameter, which reflects how uncertainty influences learning, was linked to iron concentration in the posterior cingulate cortex (PCC), precuneus, basal ganglia, and angular gyrus. The PCC is a core hub within the default mode network, involved in integrating sensory, emotional, and cognitive aspects of pain perception (*58*), and plays a role in modulating pain under uncertainty (*59*). The precuneus integrates self-referential, sensory signals, and cognitive processing (*60*), whereas the caudate and putamen, in the basal ganglia, track prediction errors in reinforcement learning (*61*, *62*). The angular gyrus is a key region for multisensory integration (*63*), with a key role in integrating conflicting sensory signals and resolving uncertainty.

The ζ parameter, which reflects individual variability in decision-making (i.e., decision temperature), was associated with iron concentration in the precuneus, cerebellum, and brainstem. The precuneus was implicated in both learning and decision-making parameters. This dual involvement supports the idea that it is a critical hub for coordinating cognitive responses to ambiguous thermal stimuli. The cerebellum is traditionally associated with motor control, but it has also been increasingly recognized for its role in cognitive functions and decision-making (*64*). Last, brainstem regions, such as the subnucleus reticularis dorsalis, modulate pain perception through the descending pain inhibitory pathway (*65*). Together, these findings suggest that microstructural properties, like iron concentration, may influence thermosensory learning and pain perception, with implications for chronic pain, where disruptions in thermosensory processing and learning are often observed (*66*).

We also observed correlations of the UMTI parameter with the myelination and iron concentration of the inferior frontal gyrus and basolateral amygdala. These regions have previously been implicated in pain processing (*34*, *35*) or threat detection (*67*) and may play a role in linking top-down learning effects with the magnification of innocuous stimuli into painful percepts, as is the case in the TGI. However, these results did not survive the TFCE-based correction and should be treated with caution pending future replication.

In general, our analysis revealed associations between individual computational profiles and the R2* parameter, a marker sensitive to iron concentration in neurons and glia. R2* is responsive to iron accumulation in regions like the substantia nigra and locus coeruleus, which contain neuromelanin, an iron-rich pigment abundant in dopaminergic neurons. Although R2* serves as an indirect proxy for iron content rather than directly measuring dopamine neuron activity, previous studies have shown its potential to reflect the integrity of dopaminergic systems, especially under neurodegenerative conditions like Parkinson’s disease (*68*, *69*). The involvement of these iron-rich, dopamine-linked regions suggests that dopaminergic processes may play a role in thermosensory associative learning. Given that iron accumulation generally increases with age and is associated with neurodegeneration, our findings may have implications for how aging affects thermosensory learning and changes in pain sensitivity across the life span.

In summary, this study refines our understanding of human thermosensation from a Bayesian perspective and identifies how uncertainty can lead to the misinterpretation of harmless stimuli as painful, transforming objectively innocuous stimuli into a subjective experience of pain. Our findings align with prior evidence supporting Bayesian models in pain perception and learning (*27*, *28*) and extend such a framework to the domains of thermosensation and thermonociceptive illusions.

Unlike previous cue-conditioning studies on nociceptive pain [e.g., (*6*)], our use of the TGI provides information into how the brain’s handling of uncertainty modulates pain perception when sensory inputs are harmless but ambiguous. Furthermore, the complex interplay between learned expectations and the sensory experience demonstrate that uncertainty computation varies substantially between individuals. By showing that innocuous thermosensation and pain perception can be modulated by learned expectations, our study provides a computational framework that may be used to identify computational profiles that might be linked to an increased vulnerability to chronic pain conditions, where pain perception is decoupled from nociceptive inputs. For instance, under neuropathic pain conditions, often marked by nerve damage and resultant sensory disruption, individuals might experience heightened pain due to a misinterpretation of uncertain thermosensory inputs. Alternatively, chronic pain might be a result of the brain’s tendency to maintain its expectation of pain, which is not updated by sensory inputs due to the reduced sensory drive. These scenarios highlight how uncertainty and expectations can shape the experience of pain, as a result of altered bottom-up signaling. Overall, our study offers a computational framework to investigate how individual differences in learning could underlie experiences of chronic pain.

## MATERIALS AND METHODS

### Participants

A total of 273 participants completed a behavioral session of the PTL task. We excluded six participants from the analyses due to missing responses in more than 10% of choices (i.e., predictions) or VAS ratings. The sample included in the behavioral analyses corresponded to 267 (182 female) participants, between the age of 18 and 52 years (mean = 24.5, SD = 4.4). A total of 213 of these participants completed an MRI session, on a separate day, prior to the completion of the PTL task. Both MRI and behavioral sessions were completed within a three-week interval by the same individuals. This research, a subsection of a larger neuroimaging study with a total of 502 participants, involved various imaging, physiological, and cognitive assessments, focusing here on thermosensory learning and qMRI data. Participants provided informed consent prior to the beginning of the study. The project received ethical approval from the Midtjylland Ethics Committee and was conducted in accordance with the Declaration of Helsinki (2013).

### Stimuli

Thermal stimuli were administered via a thermal cutaneous stimulator on the nondominant forearm, allowing for quick and accurate responses with the dominant hand. The total stimulation surface covered 10 cm^2^, comprising five distinct stimulation zones measuring 7 mm by 28 mm. Cold and warm temperatures were individually calibrated, using a procedure that combined the method of limits and method of levels approaches. Innocuous warm stimuli involved three adjacent zones at an average temperature of 39.1° ± 2.8°C, whereas innocuous cold stimuli consisted of two adjacent zones at an average temperature of 20° ± 6.5°C. The inactive zones remained at the baseline temperature of 32°C. TGI stimuli used the same temperatures as the innocuous conditions, with three warm and two cold stimuli presented in an alternating spatial pattern. Auditory tones, which served as cues for the forthcoming thermal stimulation, were either a lower tone of 400 Hz or a higher tone of 1600 Hz.

### Experimental procedure

The PTL was implemented in Matlab using the psychtoolbox-3 (*70*, *71*), where participants completed 306 trials. Each trial began with a fixation point displayed for a random interval between 1 and 2 s. Following this, an auditory tone (either 400 or 1600 Hz) was presented, leading to a prompt for the participant to predict the upcoming thermosensory stimulus. This prediction was a binary choice (i.e., “cold” or “warm”) made using the left and right arrow keys, with a response time limit of 3 s. The stimulus, lasting 10 s, varied between cold (43% of trials), warm (43% of trials), or a combination of both in an alternating pattern to induce the TGI, present in 14% of trials. In around 47% of trials, we collected VAS ratings from participants to measure their perception of cold, warm, and burning sensations. Each sensation was rated on a separate scale ranging from 0 (no sensation) to 100 (maximum sensation), with these ratings required for ~60% of cold and warm trials and all TGI trials. Participants had up to 5 s to provide each VAS rating.

The likelihood of cue-stimulus associations was governed by two predetermined sequences, which were counterbalanced across participants. These sequences were designed to create blocks of stimuli where a specific cue had an 82% chance of indicating a particular outcome. However, these cues were subject to unpredictable reversals. At certain points, a cue that previously had an 82% likelihood of predicting one outcome would switch to having only an 18% likelihood of predicting that same outcome. Interspersed between these reversals were blocks of trials in which the association between a cue and a stimulus was at chance level.

### Statistical modeling

The modeling of error rates, response times, and VAS ratings was conducted using generalized linear mixed effects models using the GAMLSS package (*72*) in R. Error rates were modeled using a binomial distribution with the logit link function, whereas response times were modeled using a gamma distribution with a logarithmic link function, VAS ratings were modeled using the zero-one-inflated beta distribution with the logit link function for all parameters (see Supplementary Note). In all mixed effects models, random intercepts were included for each subject, with random slopes added where model convergence allowed.

### Computational modeling

We compared four computational learning models in MATLAB (R2021a) (*73*): two-level HGF, Rescorla-Wagner, Sutton K1, and Pearce-Hall. To ensure the robustness of all models, we demonstrated acceptable parameter recovery, across a wide range of subject-specific learning parameters, as well as effective model recovery. Results from these analyses, further elaborated in figs. S1 to S8 ensured that the parameters derived from the models were interpretable and sensible, and facilitated the specification of reasonable parameter ranges for weakly informative priors for all models. For selecting the model that most accurately represented the data from our learning task, we used a random effects model comparison using the VBA toolbox (*54*). This analysis indicated that the two-level HGF was the most appropriate model to describe our data.

The two-level HGF uses variational Bayesian approximation to derive update equations, enabling the estimation of how beliefs across different hierarchical levels evolve over trials (*48*, *49*). The model is structured in two subparts known as response and perceptual models (Fig. 3A). The response model includes observed values, such as the inferred cue-outcome association (*U*), the participant’s prediction responses (Resp), and the decision temperature parameter zeta (ζ). The perceptual model is organized across two distinct hierarchical levels. At the first level (*x*_1_), the model captures participants’ immediate predictions about upcoming stimuli. These lower-level predictions follow a Bernouli distribution where the more extreme values around zero and one signify a low prediction uncertainty, whereas intermediate values (0.5) indicate high uncertainty. The second level (*x*_2_) encapsulated predictions about the stability of cue-outcome associations. These higher-level beliefs evolve over time as a Gaussian random walk, with a step size determined by the parameter omega (ω). The transition from the second level to the first level of the HGF is governed by a sigmoid transformation, converting the continuous Gaussian-distributed beliefs into Bernoulli-distributed probabilities about immediate outcomes. The sigmoid transformation is formulated as

$$S\left(x\right)=\frac{1}{1+e^{-x}}$$

Simultaneously, the second-level HGF is updated based on precision-weighted prediction errors computed at the first level. This update mechanism allows the model to account for confidence in the first-level predictions, adjusting the second-level beliefs accordingly. In cases of higher precision, when the uncertainty weight on prediction errors is low, the model is more likely to maintain its current belief structure. This belief updating is formulated as

$$\underset{\text{new}\;\text{prediction}}{\underbrace{\mu_{2}^{t}}}=\underset{\text{last prediction}}{\underbrace{\mu_{2}^{t-1}}}+\underset{\text{inverse precision}}{\underbrace{\frac{1}{\pi_{2}^{t}}}}\underset{\text{prediction error}}{\underbrace{\left[u^{t}-S\left(u_{2}^{t-1}\right)\right]}}$$

Furthermore, on each trial the precision is updated based on the following equation

$$\pi_{2}^{t}=\pi_{2}^{t-1}+\frac{1}{\pi_{1}^{t-1}}$$

where subscripts represent the level of the HGF, μ is the mean of the level, π is the precision of the level, *u* is the contingency input, and *t* is the trial. In our computational analysis, this inverse precision is referred to as estimation uncertainty, whereas the first-level uncertainty is labeled prediction uncertainty.

### Multiparameter mapping

We used MPM, a well-established qMRI protocol to map percent saturation due to MT, R1, and R2* (*56*, *74*, *75*). Following data acquisition, we applied VBQ (*76*) to relate individual differences in computational parameters of thermosensory learning to patterns of brain microstructure. Identical details about data acquisition and map creation are also reported in a different paper relating individual differences in respiroception to the brain microstructure (*77*). Advantages of MPM and VBQ compared to qualitative brain metrics, such as voxel-based morphometry, are improved neurobiological specificity, reproducibility, and identification of biomarkers, as well as longitudinal test-retest reliability (*78*), enhancing our understanding of brain-behavior relations.

### Data acquisition

The imaging data were acquired using a 3-T MRI scanner (Magnetom Prisma, Siemens Healthcare, Erlangen, Germany), with a standard 32-channel radio frequency (RF) head coil and a body coil. We acquired a set of high-resolution whole-brain T1-weighted anatomical images (0.8-mm isotropic) using an MP-RAGE (Magnetization Prepared – RApid Gradient Echo) sequence [repetition time (TR) = 2.2 s, echo time = 2.51 ms, matrix size = 256 × 256 × 192 voxels, flip angle = 8°, in-slice phase-encoding direction = anterior-posterior (AP), slice encoding direction = left-right (LR), and readout direction = head-foot (HF)]. Furthermore, we obtained whole-brain images at an isotropic 0.8-mm resolution using an MPM quantitative imaging protocol (*74*). The imaging sequences included three RF and gradient spoiled (using a linear phase increment of 137°) multiecho three-dimensional fast low-angle shot (FLASH) acquisitions and three additional calibration sequences to correct for RF receive bias (*79*). Specifically, the FLASH sequences consisted of MT, proton density (PD), and T1 weighting acquisitions. The flip angle was 6° for MT and PD, whereas it was 21° for T1-weighted images. MT weighting used a Gaussian RF pulse 2 kHz off resonance with 4-ms duration and a nominal flip angle of 220°. The field of view was 256-mm HF, 224-mm AP, and 179-mm RL. We acquired gradient echoes with alternating readout gradient polarity using equidistant echo times ranging from 2.34 to 13.8 ms (MT) or 18.4 ms (PD and T1), using a readout bandwidth of 490 Hz/pixel. For the MT-weighted acquisition, only six echoes were collected to achieve a TR of 25 ms for all FLASH volumes. To accelerate data acquisition, we applied the GeneRalized Autocalibrating Partial Parallel Acquisition (GRAPPA) algorithm, with an acceleration factor of 2 in each phase encoded direction and 40 integrated reference lines. All acquisitions had a slab rotation of 30° in the sagittal plane to avoid eye-related motion artifacts in the cortex. The B1 mapping acquisition consisted of 11 measurements with the nominal flip angle ranging from 115° to 65° in 5° steps. The total scanning time for the qMRI acquisitions was ~26 min.

### Map creation

We preprocessed all qMRI images using the hMRI toolbox v. 0.5.0 (January 2023) (*80*) and SMP12 (version 12.r7771, Wellcome Trust Centre for Neuroimaging, http://fil.ion.ucl.ac.uk/spm/) to correct the raw qMRI images for spatial transmit, receive field inhomogeneities, and obtain quantitative MT, PD, R1, and R2* estimate maps. Except for enabling the correction for imperfect spoiling (*81*), the hMRI toolbox was configured using the standard settings. Before the estimation of these maps, all images were aligned to the Montreal Neurologic Institute (MNI) standard space. This processing produced four maps, each reflecting different attributes of the brain tissue microstructure: An MT saturation map is sensitive to myelin content (*82*, *83*), a PD map representing tissue water content, an R1 map reflecting microstructural tissue properties such as macromolecule content, local mobility of water molecules, and iron concentration (also related to myelination) (*80*, *84*), and an R2* map sensitive to tissue iron concentration (*85*). We analyzed three of these maps (MT, R1, and R2*) independently.

We used the unified segmentation approach (*86*) to segment MT saturation maps into probability maps of GM, white matter (WM), and cerebrospinal fluid. For this segmentation, we used tissue probability maps based on multiparameter data (*87*), without bias field correction as MT maps do not show significant bias field modulation. Subsequently, GM and WM probability maps were used for intersubject registration using the Diffeomorphic Anatomical Registration Through Exponentiated Lie Algebra (DARTEL) algorithm (*88*). This step enabled the normalization of the derived quantitative maps to the MNI space at an isotropic 1-mm resolution. This normalization used the DARTEL template created during registration and participant-specific deformation fields.

The nonlinear registration of the quantitative maps was based on the MT maps, chosen for their high contrast in subcortical structures and a WM-GM contrast in the cortex comparable to that of T1-weighted images (*82*). Last, tissue-weighted smoothing was applied using a 4-mm full width at half maximum kernel (*76*) to preserve quantitative values. The resulting smoothed, modulated, and normalized GM images were used for statistical analyses. For visualization purposes, we generated average MT, R1, and R2* maps in the standard space based on data from 442 individuals, who participated in the larger project. For analysis, we used a GM mask generated by averaging the smoothed, modulated GM segments and applying a threshold of *P*(GM) > 0.2.

### MPM quality control

We implemented a comprehensive set of quality control (QC) protocols including manual and automated procedures. These included manual scoring of raw image quality at the time of acquisition, automated processing via the MRIQC pipeline (*89*), and the application of a specially developed semiautomatic hMRI-vQC pipeline designed for quantitative neuroimaging. We additionally calculated the index of motion degradation using QUality QUantification Index (QUQUI) (*84*). The automated procedures yielded a variety of quantitative QC metrics including coregistration parameters, SD in the WM of the R2* maps, SNR (signal-to-noise ratio), and contrast-to-noise ratio (CNR) values; these were inspected via boxplots to identify extreme subjects. Following the application of MRIQC, a team of two authors (N.N. and C.S.D.) inspected all raw images and postprocessed maps including those flagged by the automated procedure. Images were graded on a scale from 0 to 3 (unusable to no issues), and any disagreements between the raters were discussed and resolved alongside a third author (M.G.A.). In the larger project, 60 of 502 individuals failed to pass these QC protocols, whereas in the subsample of the current study, no participant was excluded from the VBQ analyses due to data quality.

### VBQ analyses

We analyzed whole-brain associations between MT at each voxel and thermosensory learning using a multiple linear regression approach known as VBQ. Our key analysis comprised positive and negative *t* tests over HGF-derived computational parameters (omega, zeta, and UMTI), as well as a behaviorally derived parameter (i.e., TGI responsiveness). We included age, gender, and total intracranial volume as nuisance covariates in the regression model, following recommended procedures for computational neuroanatomy (*90*).

To robustly detect clusters of voxels correlated with the computational parameters of interest, we used TFCE (*57*). Traditional cluster-based methods typically require an arbitrary voxel-wise threshold to define clusters, which can introduce bias or affect sensitivity. TFCE addresses these limitations by continuously enhancing signal from clusters of voxels based on both their spatial extent (size) and height (intensity), without the need for predefined thresholds. This makes TFCE more sensitive to subtle effects while reducing the likelihood of false positives.

For our analysis, we applied TFCE using default hyperparameters, with an extent of *E* = 0.5 and a height of *H* = 2. A family-wise error (FWE) cluster-corrected threshold of *P* < 0.05 was used within the GM mask. For comparisons to the VBQ literature, which primarily uses a traditional clustered-based parametric approach, we also conducted a cluster-wise analysis. This approach used an FWE cluster-corrected threshold of *P* < 0.025 (Bonferroni-corrected for two one-tailed tests), based on an inclusion threshold of *P* < 0.001 (uncorrected). The results from the traditional cluster-based approach are available in the Supplementary Materials for comparison.

All statistical analyses were conducted in SPM12, whereas anatomical labels were determined using the JuBrain Anatomy Toolbox v. 3.0 (*91*).

### Map interpretation

MPM produces quantitative images that allow for detailed examination of the brain microstructure. The MT map is particularly sensitive to macromolecular content, especially myelin. MT saturation reflects the exchange of magnetization between free water protons and macromolecule-bound protons, making it a key measure of myelin density and structural integrity within brain tissue (*92*). This makes MT mapping essential for analyzing regions where changes in tissue organization or myelination are relevant.

The R1 map, which reflects R1 relaxation rates, provides valuable information about tissue composition, water content, and macromolecule presence. Higher R1 values are often linked to increased macromolecule density or reduced water mobility, which may indicate myelination or other structural characteristics of the brain (*74*). As such, R1 complements the information obtained from MT mapping.

R2*, or the effective transverse relaxation rate, is highly sensitive to local magnetic field inhomogeneities caused by variations in tissue composition, such as the presence of paramagnetic substances. This makes the R2* map particularly useful for studying tissues rich in substances like iron, as well as myelin (*85*). R2* mapping is frequently applied to analyze regions where these materials are concentrated, such as in the basal ganglia and brainstem (*93*). Given the known relationship between iron accumulation and dopaminergic activity in regions like the substantia nigra, the use of R2* also provides an indirect means of investigating the health of dopamine-related pathways, particularly under neurodegenerative conditions like Parkinson’s disease (*68*, *69*).

Together, these maps provide a comprehensive quantitative approach to characterizing the brain microstructure, offering more specific and reliable measures of tissue properties than conventional qualitative MRI methods (*56*, *74*, *75*, *78*). MPM thus plays a crucial role in studying individual variations in brain anatomy and their connection to cognitive and sensory processes.

## Supplementary Materials

### The PDF file includes:

Supplementary Results Supplementary Note Figs. S1 to S8 Legends for tables S1 to S12

## References

[1] M. J. Caterina, M. A. Schumacher, M. Tominaga, T. A. Rosen, J. D. Levine, D. Julius, The capsaicin receptor: A heat-activated ion channel in the pain pathway. Nature 389, 816–824 (1997). 9349813 10.1038/39807

[2] D. D. McKemy, W. M. Neuhausser, D. Julius, Identification of a cold receptor reveals a general role for TRP channels in thermosensation. Nature 416, 52–58 (2002). 11882888 10.1038/nature719

[3] A. M. Peier, A. Moqrich, A. C. Hergarden, A. J. Reeve, D. A. Andersson, G. M. Story, T. J. Earley, I. Dragoni, P. McIntyre, S. Bevan, A. Patapoutian, A TRP channel that senses cold stimuli and menthol. Cell 108, 705–715 (2002). 11893340 10.1016/s0092-8674(02)00652-9

[4] L. Y. Atlas, N. Bolger, M. A. Lindquist, T. D. Wager, Brain mediators of predictive cue effects on perceived pain. J. Neurosci. 30, 12964–12977 (2010). 20881115 10.1523/JNEUROSCI.0057-10.2010 PMC2966558

[5] H. L. Fields, How expectations influence pain. Pain 159, S3–S10 (2018). 30113941 10.1097/j.pain.0000000000001272

[6] M. Jepma, L. Koban, J. van Doorn, M. Jones, T. D. Wager, Behavioural and neural evidence for self-reinforcing expectancy effects on pain. Nat. Hum. Behav. 2, 838–855 (2018). 31558818 10.1038/s41562-018-0455-8 PMC6768437

[7] M. M. Nickel, L. Tiemann, V. D. Hohn, E. S. May, C. Gil Ávila, F. Eippert, M. Ploner, Temporal–spectral signaling of sensory information and expectations in the cerebral processing of pain. Proc. Natl. Acad. Sci. U.S.A. 119, e2116616119 (2022). 34983852 10.1073/pnas.2116616119 PMC8740684

[8] A. D. Craig, M. C. Bushnell, The thermal grill illusion: Unmasking the burn of cold pain. Science 265, 252–255 (1994). 8023144 10.1126/science.8023144

[9] F. Fardo, N. B. Finnerup, P. Haggard, Organization of the thermal grill illusion by spinal segments. Ann. Neurol. 84, 463–472 (2018). 30063258 10.1002/ana.25307 PMC6175302

[10] F. Fardo, B. Beck, M. Allen, N. B. Finnerup, Beyond labeled lines: A population coding account of the thermal grill illusion. Neurosci. Biobehav. Rev. 108, 472–479 (2020). 31783059 10.1016/j.neubiorev.2019.11.017

[11] R. A. Böhme, L. Banellis, M. Vejlø, M. Allen, F. Fardo, The psychological construction of pain: Evidence for no link between mental health symptoms and thermal pain thresholds (2024); 10.31234/osf.io/bc627.

[12] C. Sardeto Deolindo, J. F. Ehmsen, A. S. Courtin, A. G. Mitchell, C. E. Kraenge, N. Nikolova, M. G. Allen, F. Fardo, Assessing individual sensitivity to the thermal grill illusion: A two-dimensional adaptive psychophysical approach. J. Pain 27, 104732 (2025). 39542193 10.1016/j.jpain.2024.104732

[13] A. G. Mitchell, J. F. Ehmsen, D. E. Christensen, A. V. Stuckert, P. Haggard, F. Fardo, Disentangling the spinal mechanisms of illusory heat and burning sensations in the thermal grill illusion. Pain 165, 2370–2378 (2024). 39185673 10.1097/j.pain.0000000000003352

[14] A. Leung, S. Shukla, E. Li, J.-R. Duann, T. Yaksh, Supraspinal characterization of the thermal grill illusion with fMRI. Mol. Pain 10, 18 (2014). 24612493 10.1186/1744-8069-10-18 PMC3995740

[15] A. D. Craig, E. M. Reiman, A. Evans, M. C. Bushnell, Functional imaging of an illusion of pain. Nature 384, 258–260 (1996). 8918874 10.1038/384258a0

[16] F. Lindstedt, B. Johansson, S. Martinsen, E. Kosek, P. Fransson, M. Ingvar, Evidence for thalamic involvement in the thermal grill illusion: An fMRI study. PLOS ONE 6, e27075 (2011). 22096519 10.1371/journal.pone.0027075 PMC3214046

[17] C. Büchel, S. Geuter, C. Sprenger, F. Eippert, Placebo analgesia: A predictive coding perspective. Neuron 81, 1223–1239 (2014). 24656247 10.1016/j.neuron.2014.02.042

[18] F. Fardo, R. Auksztulewicz, M. Allen, M. J. Dietz, A. Roepstorff, K. J. Friston, Expectation violation and attention to pain jointly modulate neural gain in somatosensory cortex. Neuroimage 153, 109–121 (2017). 28341164 10.1016/j.neuroimage.2017.03.041 PMC5460976

[19] S. Geuter, S. Boll, F. Eippert, C. Büchel, Functional dissociation of stimulus intensity encoding and predictive coding of pain in the insula. eLife 6, e24770 (2017). 28524817 10.7554/eLife.24770 PMC5470871

[20] A. Tabor, M. A. Thacker, G. L. Moseley, K. P. Körding, Pain: A statistical account. PLOS Comput. Biol. 13, e1005142 (2017). 28081134 10.1371/journal.pcbi.1005142 PMC5230746

[21] R. Hoskin, C. Berzuini, D. Acosta-Kane, W. El-Deredy, H. Guo, D. Talmi, Sensitivity to pain expectations: A Bayesian model of individual differences. Cognition 182, 127–139 (2019). 30243037 10.1016/j.cognition.2018.08.022

[22] B. Seymour, F. Mancini, Hierarchical models of pain: Inference, information-seeking, and adaptive control. Neuroimage 222, 117212 (2020). 32739554 10.1016/j.neuroimage.2020.117212

[23] Y. Song, M. Yao, H. Kemprecos, A. Byrne, Z. Xiao, Q. Zhang, A. Singh, J. Wang, Z. S. Chen, Predictive coding models for pain perception. J. Comput. Neurosci. 49, 107–127 (2021). 33595765 10.1007/s10827-021-00780-x PMC8046732

[24] A.-L. Eckert, K. Pabst, D. M. Endres, A Bayesian model for chronic pain. Front. Pain Res. 3, 966034 (2022). 10.3389/fpain.2022.966034 PMC9595216 36303889

[25] J. Kiverstein, M. D. Kirchhoff, M. Thacker, An embodied predictive processing theory of pain experience. Rev. Philos. Psychol. 13, 973–998 (2022).

[26] Z. S. Chen, J. Wang, Pain, from perception to action: A computational perspective. iScience 26, 105707 (2023). 36570771 10.1016/j.isci.2022.105707 PMC9771728

[27] F. Mancini, S. Zhang, B. Seymour, Computational and neural mechanisms of statistical pain learning. Nat. Commun. 13, 6613 (2022). 36329014 10.1038/s41467-022-34283-9 PMC9633765

[28] D. Mulders, B. Seymour, A. Mouraux, F. Mancini, Confidence of probabilistic predictions modulates the cortical response to pain. Proc. Natl. Acad. Sci. U.S.A. 120, e2212252120 (2023). 36669115 10.1073/pnas.2212252120 PMC9942789

[29] M. Roy, D. Shohamy, N. Daw, M. Jepma, G. E. Wimmer, T. D. Wager, Representation of aversive prediction errors in the human periaqueductal gray. Nat. Neurosci. 17, 1607–1612 (2014). 25282614 10.1038/nn.3832 PMC4213247

[30] S. Fazeli, C. Büchel, Pain-related expectation and prediction error signals in the anterior insula are not related to aversiveness. J. Neurosci. 38, 6461–6474 (2018). 29934355 10.1523/JNEUROSCI.0671-18.2018 PMC6705956

[31] J. D. Levine, N. C. Gordon, H. L. Fields, The mechanism of placebo analgesia. Lancet 2, 654–657 (1978). 80579 10.1016/s0140-6736(78)92762-9

[32] T. D. Wager, J. K. Rilling, E. E. Smith, A. Sokolik, K. L. Casey, R. J. Davidson, S. M. Kosslyn, R. M. Rose, J. D. Cohen, Placebo-induced changes in FMRI in the anticipation and experience of pain. Science 303, 1162–1167 (2004). 14976306 10.1126/science.1093065

[33] D. D. Price, D. G. Finniss, F. Benedetti, A comprehensive review of the placebo effect: Recent advances and current thought. Annu. Rev. Psychol. 59, 565–590 (2008). 17550344 10.1146/annurev.psych.59.113006.095941

[34] F. Eippert, U. Bingel, E. D. Schoell, J. Yacubian, R. Klinger, J. Lorenz, C. Büchel, Activation of the opioidergic descending pain control system underlies placebo analgesia. Neuron 63, 533–543 (2009). 19709634 10.1016/j.neuron.2009.07.014

[35] F. Eippert, J. Finsterbusch, U. Bingel, C. Büchel, Direct evidence for spinal cord involvement in placebo analgesia. Science 326, 404 (2009). 19833962 10.1126/science.1180142

[36] F. Benedetti, Placebo effects: From the neurobiological paradigm to translational implications. Neuron 84, 623–637 (2014). 25442940 10.1016/j.neuron.2014.10.023

[37] T. D. Wager, L. Y. Atlas, The neuroscience of placebo effects: Connecting context, learning and health. Nat. Rev. Neurosci. 16, 403–418 (2015). 26087681 10.1038/nrn3976 PMC6013051

[38] D. Anchisi, M. Zanon, A Bayesian perspective on sensory and cognitive integration in pain perception and placebo analgesia. PLOS ONE 10, e0117270 (2015). 25664586 10.1371/journal.pone.0117270 PMC4321992

[39] A. Tabor, C. Burr, Bayesian learning models of pain: A call to action. Curr. Opin. Behav. Sci. 26, 54–61 (2019).

[40] G. Ongaro, T. J. Kaptchuk, Symptom perception, placebo effects, and the Bayesian brain. Pain 160, 1–4 (2019). 30086114 10.1097/j.pain.0000000000001367 PMC6319577

[41] R. Kanai, B. Bahrami, G. Rees, Human parietal cortex structure predicts individual differences in perceptual rivalry. Curr. Biol. 20, 1626–1630 (2010). 20727757 10.1016/j.cub.2010.07.027 PMC2949566

[42] A. D. Craig, Can the basis for central neuropathic pain be identified by using a thermal grill? Pain 135, 215–216 (2008). 18282660 10.1016/j.pain.2008.01.022

[43] F. Adam, P. Jouët, J.-M. Sabaté, S. Perrot, C. Franchisseur, N. Attal, D. Bouhassira, Thermal grill illusion of pain in patients with chronic pain: A clinical marker of central sensitization? Pain 164, 638–644 (2023). 35972466 10.1097/j.pain.0000000000002749

[44] H. E. M. den Ouden, J. Daunizeau, J. Roiser, K. J. Friston, K. E. Stephan, Striatal prediction error modulates cortical coupling. J. Neurosci. 30, 3210–3219 (2010). 20203180 10.1523/JNEUROSCI.4458-09.2010 PMC3044875

[45] S. Iglesias, C. Mathys, K. H. Brodersen, L. Kasper, M. Piccirelli, H. E. M. den Ouden, K. E. Stephan, Hierarchical prediction errors in midbrain and basal forebrain during sensory learning. Neuron 80, 519–530 (2013). 24139048 10.1016/j.neuron.2013.09.009

[46] A. O. de Berker, R. B. Rutledge, C. Mathys, L. Marshall, G. F. Cross, R. J. Dolan, S. Bestmann, Computations of uncertainty mediate acute stress responses in humans. Nat. Commun. 7, 10996 (2016). 27020312 10.1038/ncomms10996 PMC4820542

[47] R. P. Lawson, C. Mathys, G. Rees, Adults with autism overestimate the volatility of the sensory environment. Nat. Neurosci. 20, 1293–1299 (2017). 28758996 10.1038/nn.4615 PMC5578436

[48] C. Mathys, J. Daunizeau, K. Friston, K. Stephan, A Bayesian foundation for individual learning under uncertainty. Front. Hum. Neurosci. 5, 39 (2011). 21629826 10.3389/fnhum.2011.00039 PMC3096853

[49] C. D. Mathys, E. I. Lomakina, J. Daunizeau, S. Iglesias, K. H. Brodersen, K. J. Friston, K. E. Stephan, Uncertainty in perception and the Hierarchical Gaussian Filter. Front. Hum. Neurosci. 8, 39 (2014). 25477800 10.3389/fnhum.2014.00825 PMC4237059

[50] R. Rescorla, A. Wagner, “A theory of Pavlovian conditioning: The effectiveness of reinforcement and non-reinforcement” in *Classical Conditioning: Current Research and Theory* (Appleton-Century-Crofts, 1972).

[51] R. Sutton, Gain adaptation beats least squares?, in *Proceedings of the 7th Yale Workshop on Adaptive and Learning Systems* (Yale University, 1995).

[52] J. Li, D. Schiller, G. Schoenbaum, E. A. Phelps, N. D. Daw, Differential roles of human striatum and amygdala in associative learning. Nat. Neurosci. 14, 1250–1252 (2011). 21909088 10.1038/nn.2904 PMC3268261

[53] S. Zhang, H. Mano, G. Ganesh, T. Robbins, B. Seymour, Dissociable learning processes underlie human pain conditioning. Curr. Biol. 26, 52–58 (2016). 26711494 10.1016/j.cub.2015.10.066 PMC4712170

[54] J. Daunizeau, V. Adam, L. Rigoux, VBA: A probabilistic treatment of nonlinear models for neurobiological and behavioural data. PLOS Comput. Biol. 10, e1003441 (2014). 24465198 10.1371/journal.pcbi.1003441 PMC3900378

[55] R. S. Samson, O. Ciccarelli, C. Kachramanoglou, L. Brightman, A. Lutti, D. L. Thomas, N. Weiskopf, C. A. M. Wheeler-Kingshott, Tissue- and column-specific measurements from multi-parameter mapping of the human cervical spinal cord at 3 T. NMR Biomed. 26, 1823–1830 (2013). 24105923 10.1002/nbm.3022 PMC4034603

[56] N. Weiskopf, S. Mohammadi, A. Lutti, M. F. Callaghan, Advances in MRI-based computational neuroanatomy: From morphometry to in-vivo histology. Curr. Opin. Neurol. 28, 313–322 (2015). 26132532 10.1097/WCO.0000000000000222

[57] S. M. Smith, T. E. Nichols, Threshold-free cluster enhancement: Addressing problems of smoothing, threshold dependence and localisation in cluster inference. Neuroimage 44, 83–98 (2009). 18501637 10.1016/j.neuroimage.2008.03.061

[58] B. A. Vogt, S. Laureys, Posterior cingulate, precuneal and retrosplenial cortices: Cytology and components of the neural network correlates of consciousness. Prog. Brain Res. 150, 205–217 (2005). 16186025 10.1016/S0079-6123(05)50015-3 PMC2679949

[59] R. Leech, D. J. Sharp, The role of the posterior cingulate cortex in cognition and disease. Brain 137, 12–32 (2014). 23869106 10.1093/brain/awt162 PMC3891440

[60] A. E. Cavanna, M. R. Trimble, The precuneus: A review of its functional anatomy and behavioural correlates. Brain 129, 564–583 (2006). 16399806 10.1093/brain/awl004

[61] J. P. O’Doherty, P. Dayan, K. Friston, H. Critchley, R. J. Dolan, Temporal difference models and reward-related learning in the human brain. Neuron 38, 329–337 (2003). 12718865 10.1016/s0896-6273(03)00169-7

[62] J. O’Doherty, P. Dayan, J. Schultz, R. Deichmann, K. Friston, R. J. Dolan, Dissociable roles of ventral and dorsal striatum in instrumental conditioning. Science 304, 452–454 (2004). 15087550 10.1126/science.1094285

[63] M. L. Seghier, The angular gyrus: Multiple functions and multiple subdivisions. Neuroscientist 19, 43–61 (2013). 22547530 10.1177/1073858412440596 PMC4107834

[64] J. D. Schmahmann, The cerebellum and cognition. Neurosci. Lett. 688, 62–75 (2019). 29997061 10.1016/j.neulet.2018.07.005

[65] I. Tracey, P. W. Mantyh, The cerebral signature for pain perception and its modulation. Neuron 55, 377–391 (2007). 17678852 10.1016/j.neuron.2007.07.012

[66] A. V. Apkarian, M. N. Baliki, P. Y. Geha, Towards a theory of chronic pain. Prog. Neurobiol. 87, 81–97 (2009). 18952143 10.1016/j.pneurobio.2008.09.018 PMC2650821

[67] A. S. Fox, J. A. Oler, D. P. M. Tromp, J. L. Fudge, N. H. Kalin, Extending the amygdala in theories of threat processing. Trends Neurosci. 38, 319–329 (2015). 25851307 10.1016/j.tins.2015.03.002 PMC4417372

[68] T. K. Steiger, N. Weiskopf, N. Bunzeck, Iron level and myelin content in the ventral striatum predict memory performance in the aging brain. J. Neurosci. 36, 3552–3558 (2016). 27013683 10.1523/JNEUROSCI.3617-15.2016 PMC4804012

[69] P. Trujillo, M. A. Aumann, D. O. Claassen, Neuromelanin-sensitive MRI as a promising biomarker of catecholamine function. Brain 147, 337–351 (2024). 37669320 10.1093/brain/awad300 PMC10834262

[70] D. H. Brainard, The psychophysics toolbox. Spat. Vis. 10, 433–436 (1997). 9176952

[71] M. Kleiner, D. Brainard, D. Pelli, A. Ingling, R. Murray, C. Broussard, What’s new in psychtoolbox-3. Perception 36, 1–16 (2007).

[72] R. A. Rigby, D. M. Stasinopoulos, Generalized additive models for location, scale and shape. J. R. Stat. Soc. Ser. C. Appl. Stat. 54, 507–554 (2005).

[73] The Mathworks Inc., MATLAB version 9.10.0.1613233 (R2021a) (The Mathworks Inc., 2021).

[74] N. Weiskopf, J. Suckling, G. Williams, M. M. Correia, B. Inkster, R. Tait, C. Ooi, E. T. Bullmore, A. Lutti, Quantitative multi-parameter mapping of R1, PD*, MT, and R2* at 3T: A multi-center validation. Front. Neurosci. 7, 95 (2013). 23772204 10.3389/fnins.2013.00095 PMC3677134

[75] M. F. Callaghan, P. Freund, B. Draganski, E. Anderson, M. Cappelletti, R. Chowdhury, J. Diedrichsen, T. H. B. Fitzgerald, P. Smittenaar, G. Helms, A. Lutti, N. Weiskopf, Widespread age-related differences in the human brain microstructure revealed by quantitative magnetic resonance imaging. Neurobiol. Aging 35, 1862–1872 (2014). 24656835 10.1016/j.neurobiolaging.2014.02.008 PMC4024196

[76] B. Draganski, J. Ashburner, C. Hutton, F. Kherif, R. S. J. Frackowiak, G. Helms, N. Weiskopf, Regional specificity of MRI contrast parameter changes in normal ageing revealed by voxel-based quantification (VBQ). Neuroimage 55, 1423–1434 (2011). 21277375 10.1016/j.neuroimage.2011.01.052 PMC3093621

[77] N. Nikolova, J. F. Ehmsen, L. Banellis, M. Brændholt, M. Vejlø, F. Fardo, M. Allen, Microstructural brain correlates of inter-individual differences in respiratory interoception. bioRxiv 588519 [Preprint] (2024). 10.1101/2024.04.08.588519. PMC12410050 40750358

[78] N. Aye, N. Lehmann, J. Kaufmann, H.-J. Heinze, E. Düzel, M. Taubert, G. Ziegler, Test-retest reliability of multi-parametric maps (MPM) of brain microstructure. Neuroimage 256, 119249 (2022). 35487455 10.1016/j.neuroimage.2022.119249

[79] D. Papp, M. F. Callaghan, H. Meyer, C. Buckley, N. Weiskopf, Correction of inter-scan motion artifacts in quantitative R1 mapping by accounting for receive coil sensitivity effects. Magn. Reson. Med. 76, 1478–1485 (2016). 26608936 10.1002/mrm.26058 PMC5082493

[80] K. Tabelow, E. Balteau, J. Ashburner, M. F. Callaghan, B. Draganski, G. Helms, F. Kherif, T. Leutritz, A. Lutti, C. Phillips, E. Reimer, L. Ruthotto, M. Seif, N. Weiskopf, G. Ziegler, S. Mohammadi, hMRI—A toolbox for quantitative MRI in neuroscience and clinical research. Neuroimage 194, 191–210 (2019). 30677501 10.1016/j.neuroimage.2019.01.029 PMC6547054

[81] N. Corbin, M. F. Callaghan, Imperfect spoiling in variable flip angle T1 mapping at 7T: Quantifying and minimizing impact. Magn. Reson. Med. 86, 693–708 (2021). 33645814 10.1002/mrm.28720 PMC8436769

[82] G. Helms, B. Draganski, R. Frackowiak, J. Ashburner, N. Weiskopf, Improved segmentation of deep brain grey matter structures using magnetization transfer (MT) parameter maps. Neuroimage 47, 194–198 (2009). 19344771 10.1016/j.neuroimage.2009.03.053 PMC2694257

[83] P. Freund, N. Weiskopf, J. Ashburner, K. Wolf, R. Sutter, D. R. Altmann, K. Friston, A. Thompson, A. Curt, MRI investigation of the sensorimotor cortex and the corticospinal tract after acute spinal cord injury: A prospective longitudinal study. Lancet Neurol. 12, 873–881 (2013). 23827394 10.1016/S1474-4422(13)70146-7 PMC3744750

[84] A. Lutti, N. Corbin, J. Ashburner, G. Ziegler, B. Draganski, C. Phillips, F. Kherif, M. F. Callaghan, G. Di Domenicantonio, Restoring statistical validity in group analyses of motion-corrupted MRI data. Human Brain Mapp. 43, 1973–1983 (2022). 10.1002/hbm.25767 PMC8933245 35112434

[85] C. Langkammer, N. Krebs, W. Goessler, E. Scheurer, F. Ebner, K. Yen, F. Fazekas, S. Ropele, Quantitative MR imaging of brain iron: A postmortem validation study. Radiology 257, 455–462 (2010). 20843991 10.1148/radiol.10100495

[86] J. Ashburner, K. J. Friston, Unified segmentation. Neuroimage 26, 839–851 (2005). 15955494 10.1016/j.neuroimage.2005.02.018

[87] S. Lorio, S. Fresard, S. Adaszewski, F. Kherif, R. Chowdhury, R. S. Frackowiak, J. Ashburner, G. Helms, N. Weiskopf, A. Lutti, B. Draganski, New tissue priors for improved automated classification of subcortical brain structures on MRI. Neuroimage 130, 157–166 (2016). 26854557 10.1016/j.neuroimage.2016.01.062 PMC4819722

[88] J. Ashburner, A fast diffeomorphic image registration algorithm. Neuroimage 38, 95–113 (2007). 17761438 10.1016/j.neuroimage.2007.07.007

[89] O. Esteban, D. Birman, M. Schaer, O. O. Koyejo, R. A. Poldrack, K. J. Gorgolewski, MRIQC: Advancing the automatic prediction of image quality in MRI from unseen sites. PLOS ONE 12, e0184661 (2017). 28945803 10.1371/journal.pone.0184661 PMC5612458

[90] G. R. Ridgway, S. M. D. Henley, J. D. Rohrer, R. I. Scahill, J. D. Warren, N. C. Fox, Ten simple rules for reporting voxel-based morphometry studies. Neuroimage 40, 1429–1435 (2008). 18314353 10.1016/j.neuroimage.2008.01.003

[91] S. B. Eickhoff, K. E. Stephan, H. Mohlberg, C. Grefkes, G. R. Fink, K. Amunts, K. Zilles, A new SPM toolbox for combining probabilistic cytoarchitectonic maps and functional imaging data. Neuroimage 25, 1325–1335 (2005). 15850749 10.1016/j.neuroimage.2004.12.034

[92] G. Helms, H. Dathe, K. Kallenberg, P. Dechent, High-resolution maps of magnetization transfer with inherent correction for RF inhomogeneity and T1 relaxation obtained from 3D FLASH MRI. Magn. Reson. Med. 60, 1396–1407 (2008). 19025906 10.1002/mrm.21732

[93] M. C. Keuken, P.-L. Bazin, L. Crown, J. Hootsmans, A. Laufer, C. Müller-Axt, R. Sier, E. J. van der Putten, A. Schäfer, R. Turner, B. U. Forstmann, Quantifying inter-individual anatomical variability in the subcortex using 7 T structural MRI. Neuroimage 94, 40–46 (2014). 24650599 10.1016/j.neuroimage.2014.03.032

