# Supplementary material for: Thermosensory predictive coding underpins an illusion of pain
Source: Sci Adv. 2025 Mar 12;11(11):eadq0261. doi: 10.1126/sciadv.adq0261 (PMC11900864; doi:10.1126/sciadv.adq0261)
Supplement: Supplementary file 1 — Supplementary Results Supplementary Note Figs. S1 to S8 Legends for tables S1 to S12 [file sciadv.adq0261_sm.pdf]

Science Advances
AAAS

Supplementary Materials for

Thermosensory predictive coding underpins an illusion of pain

Jesper Fischer Ehmsen *et al.*

Corresponding author: Francesca Fardo, francesca@cfin.au.dk

Sci. Adv. **11**, eadq0261 (2025)
DOI: 10.1126/sciadv.adq0261

The PDF file includes:

Supplementary Results
Supplementary Note
Figs. S1 to S8
Legends for tables S1 to S12

Other Supplementary Material for this manuscript includes the following:

Tables S1 to S12

Supplementary material

Supplementary Results

We investigated how response times were affected in trials after stimuli designed to elicit the Thermal Grill Illusion (TGI). Our findings revealed a significant influence of the interaction between cue-stimulus association and participants' perception of TGI quality (β = 0.14, 95% CI = [0.07; 0.22], p < .0001). Specifically, when there was a congruence between the predicted temperature (contingency) and the actual perceived TGI quality (e.g., anticipating cold and perceiving the TGI as predominantly cold), participants' response times on the trial following a TGI stimulus remained unchanged, indicating no post-TGI slowing (β = -0.02, 95% CI = [-0.07; 0.02], p = .32). Conversely, when there was a mismatch between the predicted temperature and perceived TGI quality (for instance, expecting warm but perceiving TGI as predominantly cold), participants exhibited slower response times in the subsequent trial (β = 0.12, 95% CI = [0.06; 0.17], p < .0001). Further details can be found in the Supplementary Tables.

Supplementary Note

Formulation of reported models

We analyzed three types of responses: (1) binary choices, which determined if a participant predicted a cold or a warm stimulus, (2) response times associated with these binary choices and (3) VAS ratings, which reflected how a stimulus was perceived by a participant. Here, we detail the probability distribution of each response type, as well as the parameters upon which our regression analysis was based.

1. Binary choices

Binary choices were modeled via the binomial distribution:

$$f(y|\mu) = \frac{\Gamma(n+1)}{\Gamma(y+1)\Gamma(n-y+1)}\mu^{y}(1-\mu)^{(n-y)},$$

Where Γ is the gamma function, y is the observed number of "successes", and $\mu \in [0,1]$ is the success probability. Here, we parameterized μ using the logit link function (the inverse sigmoid transformation):

$$logit(\mu) = log\left(\frac{\mu}{1-\mu}\right).$$

2. Response times

Response times were modelled via the gamma distribution:

$$f(y|\mu,\sigma) = \frac{y^{(1/\sigma^2-1)}e^{[-y/(\sigma^2\mu)]}}{(\sigma^2\mu)^{(1/\sigma^2)}\Gamma(1/\sigma^2)},$$

Where Γ is the gamma function, y is the response time (restricted to positive values), $\mu \in]0,\inf[$ is the mean, and $\sigma \in]0,\inf[$ is the square root of the usual dispersion parameter from the GLM gamma model. Note that $\sigma * \mu$ is the standard deviation of the distribution. Here we used a logarithmic link function for μ.

3. **Visual Analog Scale (VAS) ratings**

VAS ratings were modeled using the zero–one inflated beta (ZOIB) distribution, which is a mixture of two Bernoulli distributions (for 0 and 1 outcomes) and one beta distribution (for values strictly between 0 and 1). Formally,

$$beinf(y|p_0, p_1, \mu, \phi) = \begin{cases} p_0 & y = 0 \\ f(y|\mu, \phi) & y \in [0,1], \\ p_1 & y = 1 \end{cases}$$

where the beta density $f(y; \mu, \phi)$ is

$$f(y; \mu, \phi) = \frac{\Gamma(\alpha+\beta)}{\Gamma(\alpha)\Gamma(\beta)} y^{\alpha-1}(1-y)^{\beta-1}, y \in [0,1].$$

In GAMLSS, these parameters are parameterized as:

$$\mu = \frac{\alpha}{\alpha+\beta},$$

$$\sigma = \frac{1}{\alpha+\beta+1},$$

$$\tau = \frac{p_0}{p_2},$$

$$\nu = \frac{p_1}{p_2},$$

where $p_2 = 1 - p_0 - p_1$.

All parameters (μ, σ, τ and ν) lie in (0,1), and are modelled using the logit link function.

Formulation of the Uncertainty Modulation of TGI Index

To provide a thorough understanding of the subject-specific Uncertainty Modulation index parameter (UMTI), here we present the detailed mathematical formulation of the model. This formulation is written using linear mixed effects syntax:

$$Burning_i \sim Est_i * Stim_i + Trial_i + (Est * Stim|ID),$$
$$family = beinf(\mu_i, \tau_i, \nu_i, \kappa)$$

Although multiple parameters can be modeled, our main focus here is on the mean component, μ. However, it is important to note that this approach is equally applicable to the parameters representing the proportion of ones and zeros (i.e., ν & τ). The mathematical description, specifically tailored to address only the mean, is as follows:

$$Burning_i \sim beinf(\mu_i, \tau, \nu, \kappa)$$
$$u_{i,j} = \beta_{0_J} + \beta_4 * Est_{i_j} + \beta_5 * Stim_{i_j} + \beta_6 * Trial_i + \beta_{1_J} * \left(Est_{i_j} * Stim(cold)_{i_j}\right) + \beta_{2_J} * \left(Est_{i_j} * Stim(warm)_{i_j}\right) + \beta_{3_J} * \left(Est_{i_j} * Stim(TGI)_{i_j}\right)$$

The random effects are modeled via a multivariate normal distribution with a variance–covariance matrix. Here, we exclude the upper triangle of the matrix to avoid redundancy.

$$\begin{pmatrix} \beta_{0_J} \\ \beta_{1_J} \\ \beta_{2_J} \\ \beta_{3_J} \end{pmatrix} \sim \mathcal{N}\left(\begin{pmatrix} \mu_{\beta_0} \\ \mu_{\beta_1} \\ \mu_{\beta_2} \\ \mu_{\beta_3} \end{pmatrix}, \begin{bmatrix} \sigma_{\beta_0}^2 & . & . & . \\ \sigma_{\beta_0}\sigma_{\beta_1}\rho_1 & \sigma_{\beta_1}^2 & . & . \\ \sigma_{\beta_0}\sigma_{\beta_2}\rho_2 & \sigma_{\beta_1}\sigma_{\beta_2}\rho_4 & \sigma_{\beta_2}^2 & . \\ \sigma_{\beta_0}\sigma_{\beta_3}\rho_3 & \sigma_{\beta_1}\sigma_{\beta_3}\rho_5 & \sigma_{\beta_2}\sigma_{\beta_3}\rho_6 & \sigma_{\beta_3}^2 \end{bmatrix} \right)$$

Our parameter of interest (i.e., UMTI) is β_{3_j}. This term quantifies how estimation uncertainty ("Est") modulates the burning rating for TGI stimuli relative to cold or warm stimuli. A positive β_{3_j} implies that a participant's burning ratings increase more steeply for TGI stimuli (compared to cold or warm) as estimation uncertainty increases. This effect is distinct from the direct impact of the TGI stimulus itself; it represents the differential impact of estimation uncertainty on burning ratings across stimulus types.

Multi-Parameter Mapping

In our initial analysis, we identified correlations between multi-parameter maps and the computational parameters of interest using a traditional cluster-based inference approach. This approach applied a family-wise error (FWE) cluster-corrected threshold of $p < 0.025$ (Bonferroni-corrected for two one-tailed tests), with an inclusion threshold of $p < 0.001$ (uncorrected) within the gray matter mask. The regression model included the computational parameters omega, zeta, and UMTI, along with age, gender, and total intracranial volume (TIV) as nuisance covariates. These results were initially reported in the preprint version of the manuscript (version 1) and are available online in the associated zenodo and github.

In response to a reviewer's suggestion, we updated the model to include TGI responsiveness as an additional regressor of interest. For this updated analysis, we performed both the original traditional cluster-based inference and Threshold-Free Cluster Enhancement (TFCE). Given that TFCE offers key advantages over traditional methods (such as enhanced sensitivity to subtle effects and the avoidance of arbitrary cluster-forming thresholds), we updated the main manuscript's methods and results sections to reflect the findings obtained using TFCE. Nevertheless, for completeness and comparison, we also provide the results from the traditional cluster-based inference method in an online repository in the associated zenodo and github.

Supplementary Figures

To ensure the robustness of our models, we conducted parameter recovery analysis. This analysis revealed that the 3-level Hierarchical Gaussian Filter model and the modified Pearce-Hall model could not adequately recover all the parameters governing the learning trajectories. Consequently, these models were not included in neither model comparison nor model selection.

The parameter recovery analysis demonstrated that the 2-level HGF, the Rescorla-Wagner, the Sutton k1 and the Pearce-Hall learning models successfully recovered their respective parameters with acceptable precision. However, the 3-level HGF and the modified Pearce-Hall failed to recover particular parameters, making it unsuitable for further analysis in this context. The outcomes of the parameter recovery were then utilized to establish suitable priors for subsequent model recovery analyses. For further details, including comprehensive plots that illustrate the evaluation of the priors used in our simulations, readers are directed to the Shiny app in the GitHub repository linked to this study.

Note we display $\mathcal{N}(\mu, \sigma^2)$ as the HGF toolbox. All parameters were simulated from uniform distributions over the ranges indicated in the respective figures. For clarity, the parameter-recovery figures for ζ are truncated on the y-axis at 30; a small number of simulations estimated ζ values above 50, which were omitted in the scatter plots but included in the reported correlation coefficients. Priors in each model were transformed to respect parameter constraints, meaning parameters on the unit interval were sigmoid transformed, and positively constrained parameters were exponentiated.

Fig S1.

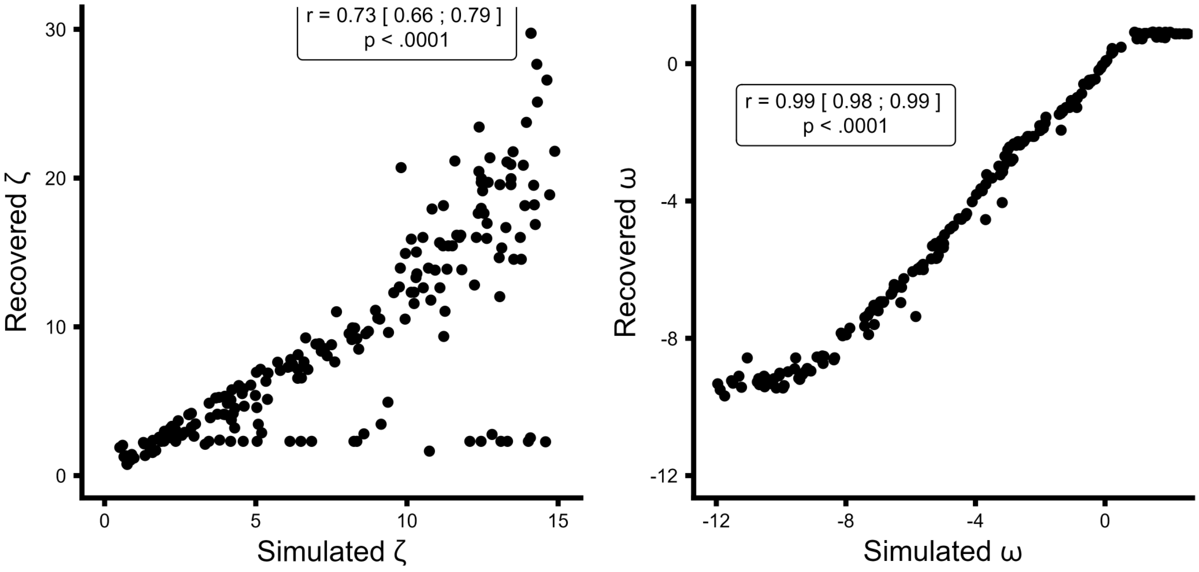

Parameter recovery analysis of the 2-level Hierarchical Gaussian Filter learning model. Each scatter plot shows simulated parameter values (x-axis) versus the recovered (estimated) values (y-axis) for ζ (left) and ω (right). Pearson correlation coefficients (r) and their 95% confidence intervals quantify the alignment between simulated and recovered values. Priors were specified as $\omega \sim N(-3,16)$ and $\zeta \sim N(5,3)$.

Fig S2.

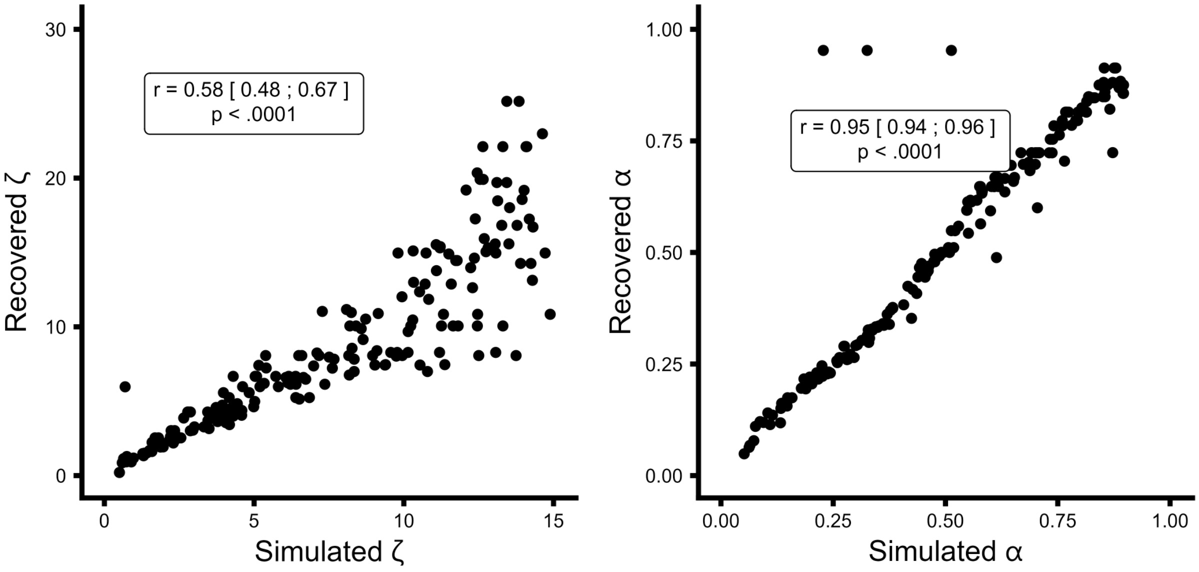

Parameter recovery analysis of the Rescorla-Wagner learning model. Each scatter plot shows the simulated parameter values (x-axis) against the recovered (estimated) values (y-axis) for ζ (left) and α (right). Reported correlation coefficients (r) and 95% confidence intervals quantify the alignment between the true and recovered values. Priors were specified as $\alpha \sim N(0,2)$ and $\zeta \sim N(5,3)$.

Fig S3.

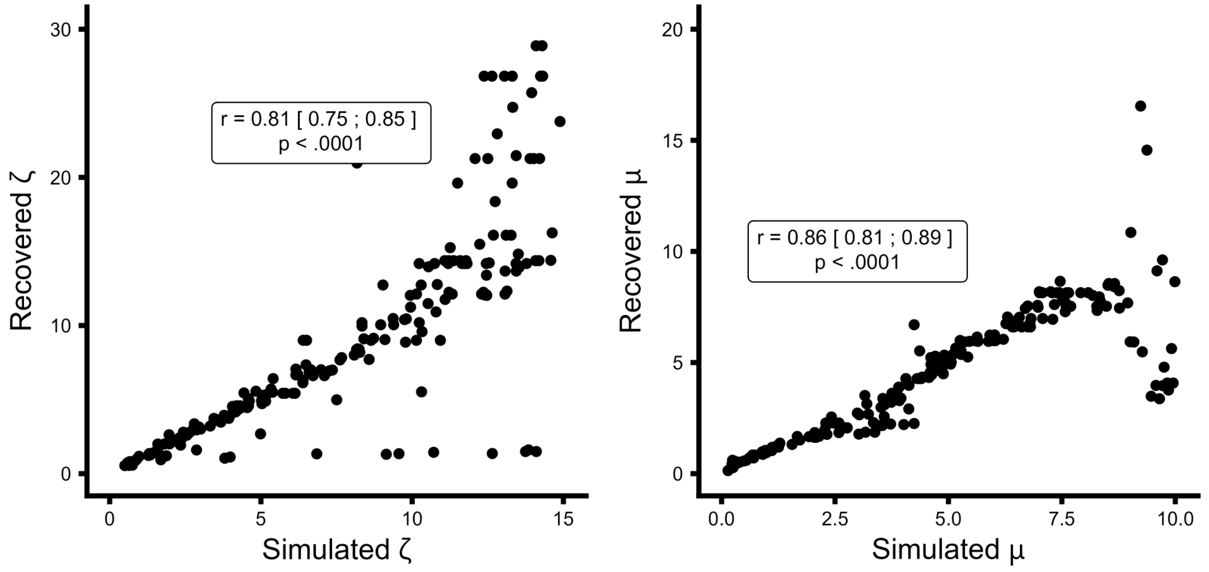

Parameter recovery analysis of the Sutton K1 learning model. Each scatter plot shows the simulated parameter values on the x-axis against the recovered (estimated) values on the y-axis for ζ (left) and μ (right). Pearson correlation coefficients (r) and 95% confidence intervals quantify the alignment between true and recovered values. Priors were set as $\mu \sim N(3,10)$ and $\zeta \sim N(5,3)$.

Fig S4.

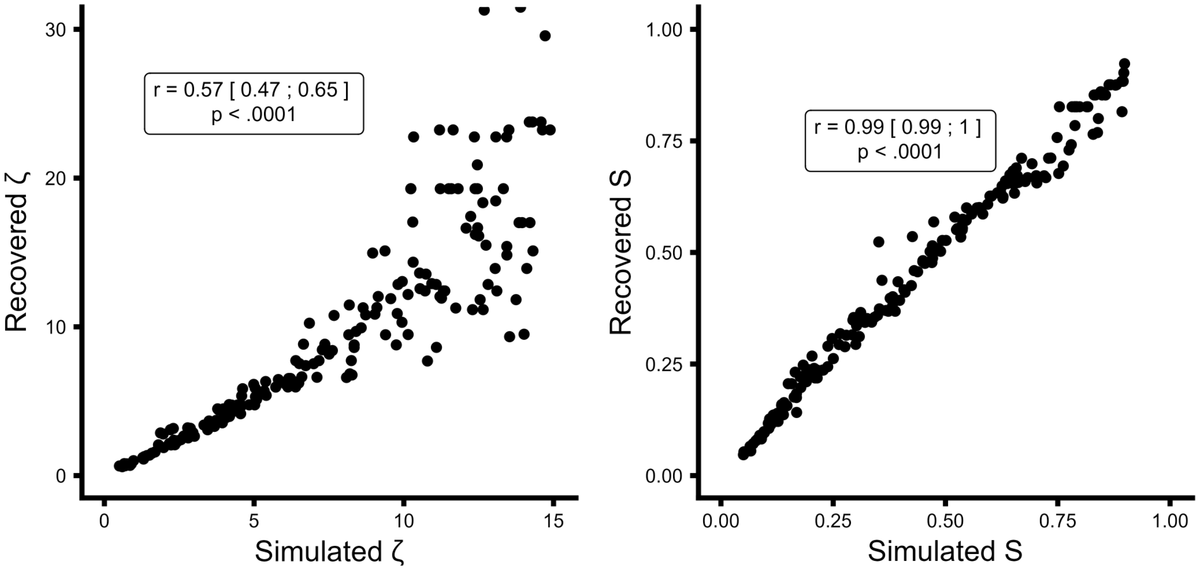

Parameter recovery analysis of the Pearce-Hall learning model. Each scatter plot shows the simulated parameter values (x-axis) versus the recovered (estimated) values (y-axis) for ζ (left) and S (right). Reported correlation coefficients (r) and their 95% confidence intervals indicate the alignment between true and recovered values. Priors were set as S ~ $N(0,2)$ and ζ ~ $N(5,3)$.

Fig S5.

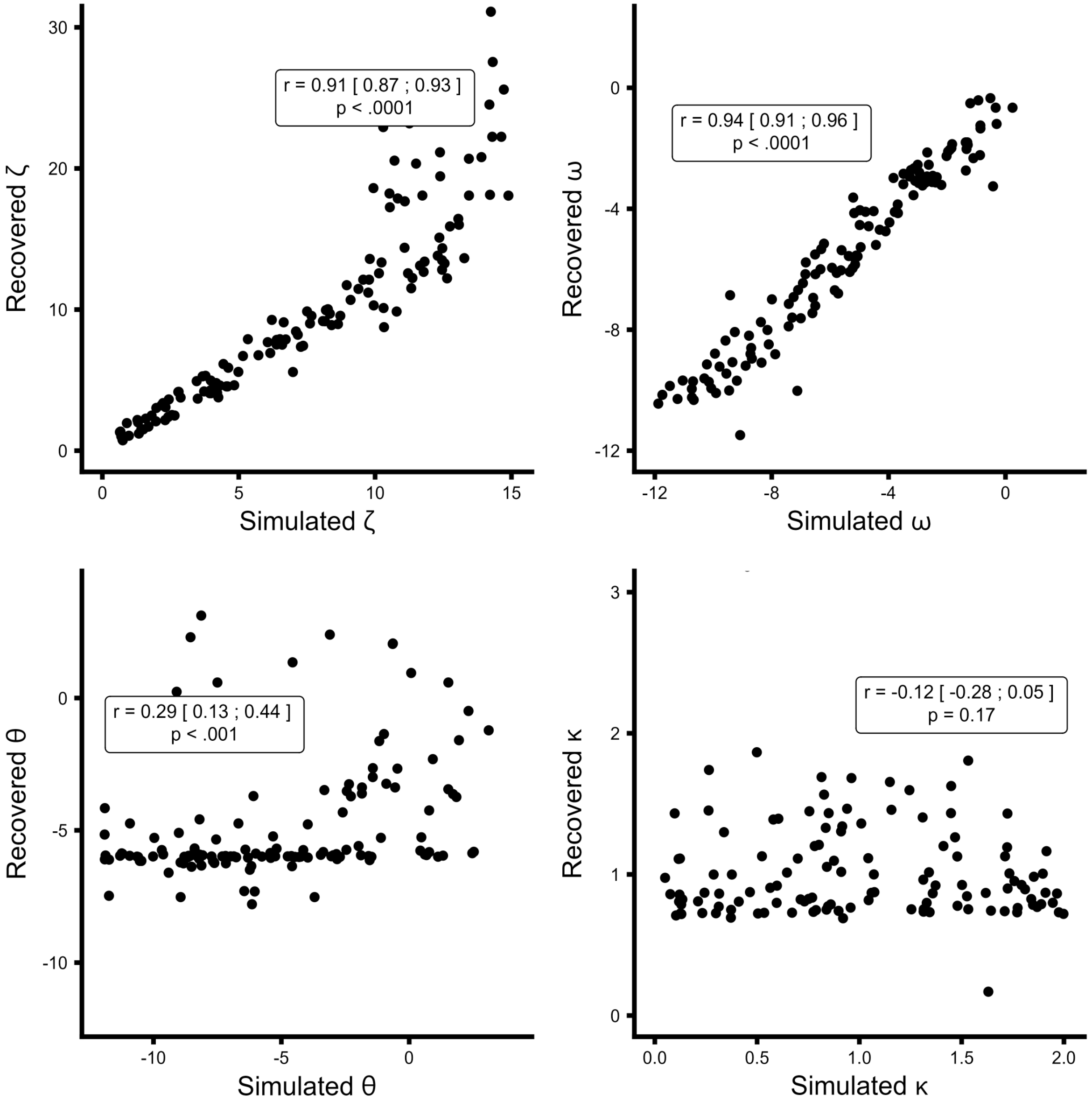

Parameter recovery analysis of the 3-level Hierarchical Gaussian Filter learning model. Each scatter plot shows the simulated parameter values (x-axis) versus the recovered (estimated) values (y-axis) for ζ (top left), ω (top right), θ (bottom left), and κ (bottom right). Pearson correlation coefficients (r) and 95% confidence intervals evaluate the alignment between the true and recovered values. Priors for all parameters were specified as follows: $\omega \sim N(-3,16)$, $\zeta \sim N(5,3)$, $\theta \sim N(-6,16)$, and $\kappa \sim N(1,1)$. Owing to the poor recovery of the third-level parameters (θ and κ), the three-level HGF model was excluded from further model comparisons.

Fig S6.

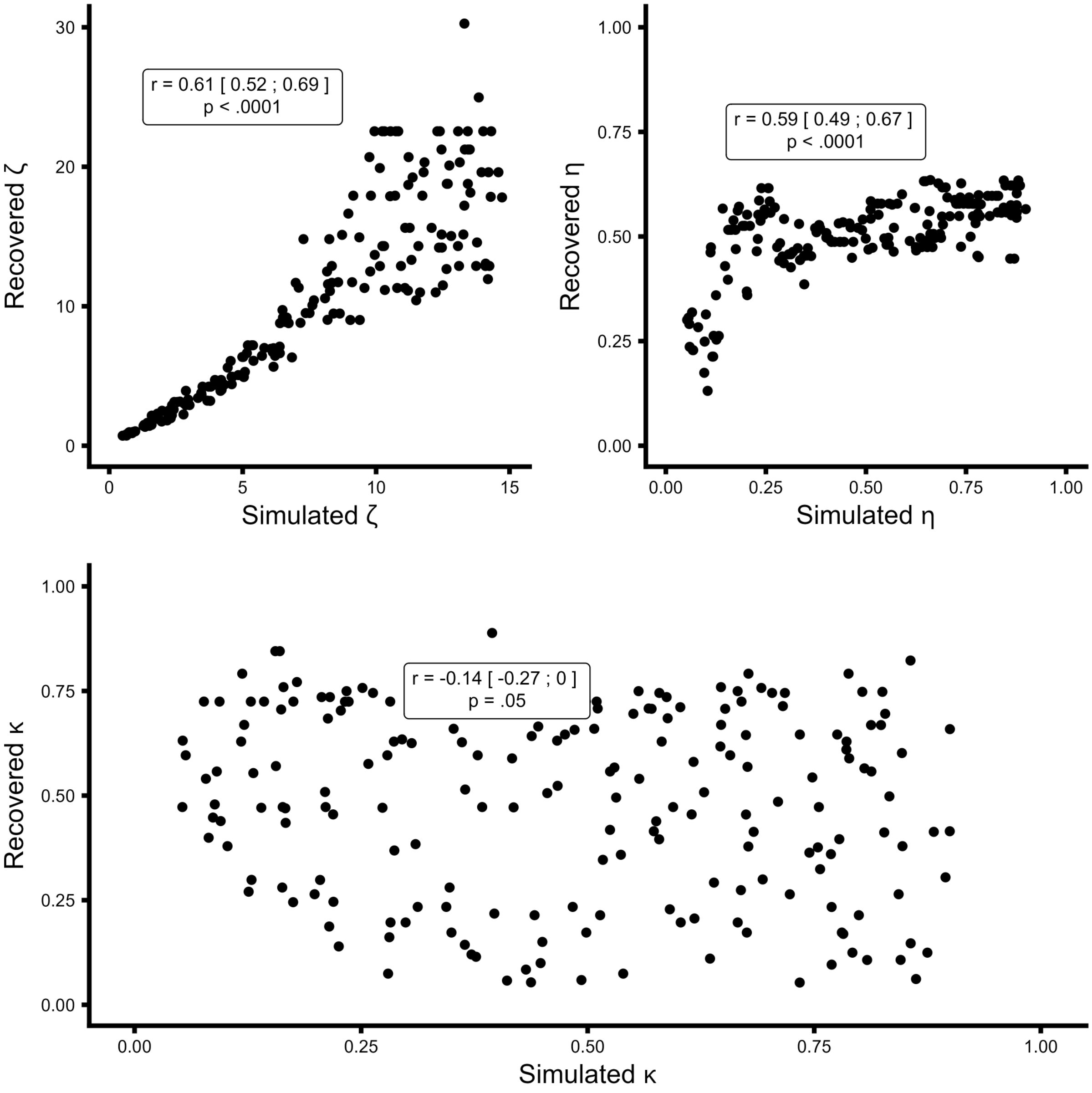

Parameter recovery analysis of the modified Pearce–Hall learning model. Each scatter plot shows simulated parameter values on the x-axis versus the corresponding recovered (estimated) values on the y-axis for (top-left) ζ, (top-right) η, and (bottom) κ. The reported correlation (r) and its 95% confidence interval assess how closely recovered estimates align with the true simulated values. Priors were set as follows: $\kappa \sim N(0,2)$, $\zeta \sim N(5,3)$, and $\eta \sim N(0,2)$. Because κ showed very poor recoverability, the modified Pearce–Hall model was excluded from subsequent model comparison analysis.

Fig S7.

		Simulated			
		**HGF**	**RW**	**SU1**	**PH**
**Recovered**	**HGF**	186	0	35	4
	**RW**	1	195	0	0
	**SU1**	8	0	153	0
	**PH**	5	5	12	196

Model recovery analysis. Columns correspond to the model used for data generation (Simulated), while rows represent the model that provided the best fit to those data in terms of log model evidence (Recovered). The high counts along the diagonal indicate that each generative model is most often recovered by itself, demonstrating that these models are sufficiently distinct to be reliably identified.

The model recovery analysis is based on the following priors:

- **HGF:** $\omega \sim N(-4,6)$ & $\zeta \sim N(5,2)$
- **Rescorla-Wagner (RW):** $\alpha \sim N(0,1)$ & $\zeta \sim N(5,3)$
- **Sutton K1 (SU1):** $\mu \sim N(3,10)$ & $\zeta \sim N(5,3)$
- **Pearce-Hall (PH):** $S \sim N(0,1)$ & $\zeta \sim N(5,3)$

Fig S8.

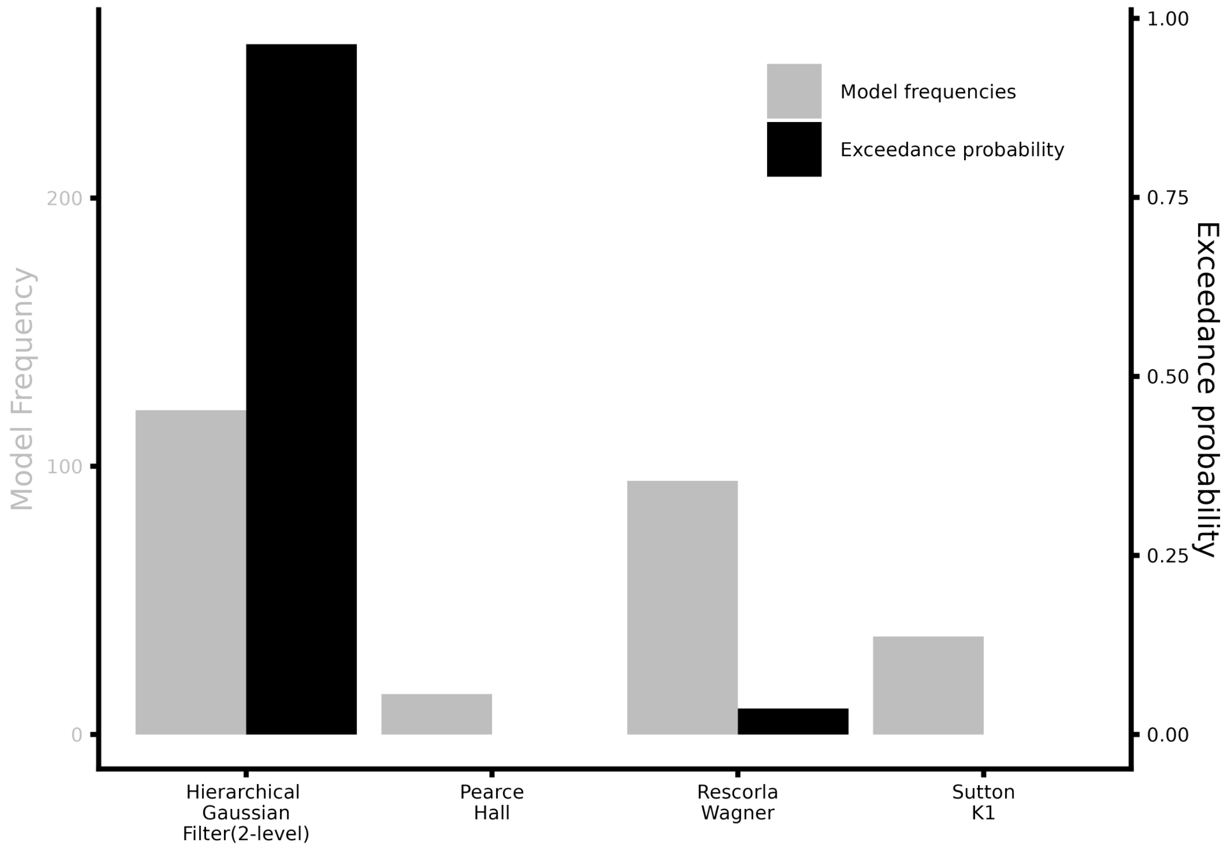

Model selection analysis using random-effects Bayesian model comparison. Gray bars denote posterior model frequencies, while black bars represent exceedance probabilities for each of the four models tested: the two-level Hierarchical Gaussian Filter, Pearce–Hall, Rescorla–Wagner, and Sutton K1. The two-level Hierarchical Gaussian Filter outperformed the other models.

Supplementary Tables

Table S1. Main effect of expectation on next-stimulus prediction.

Table S2. Main effect of expectation on next-stimulus response time.

Table S3. Main effect of stimulus on burning ratings.

Table S4. Main effect of stimulus on cold ratings.

Table S5. Main effect of stimulus on warm ratings.

Table S6. Effect of expectation on thermosensory ratings.

Table S7. Effect of perceived TGI, given contingency, on next-trial accuracy.

Table S8. Effect of perceived TGI, given contingency, on next-trial prediction response time.

Table S9. Effect of prediction uncertainty on current-trial accuracy.

Table S10. Effect of prediction uncertainty on current-trial prediction time.

Table S11. Effect of belief that the next stimulus will be cold on thermosensory ratings.

Table S12. Effect of estimation uncertainty, given stimulus, on burning ratings.
